# Transport–Reaction–Signal Coupling in Lateral Flow Assays for Next‐Generation Point‐of‐Care Diagnostics

**DOI:** 10.1002/advs.77916

**Published:** 2026-09-30

**Authors:** Yan Pan, Yuewei Li, Chao Mi, Zhengwen Yang, Lana McClements, Steven J. Langford, Jiayan Liao

**Affiliations:** ^1^ Institute For Biomedical Materials and Devices (IBMD) Faculty of Science University of Technology Sydney Ultimo New South Wales Australia; ^2^ School of Mathematical and Physical Sciences Faculty of Science University of Technology Sydney Ultimo New South Wales Australia; ^3^ School of Advanced Engineering Great Bay Institute for Advanced Study Great Bay University Dongguan Guangdong China; ^4^ College of Materials Science and Engineering Kunming University of Science and Technology Kunming China; ^5^ School of Life Sciences Faculty of Science University of Technology Sydney Sydney New South Wales Australia

**Keywords:** artificial intelligence, fluid dynamics, immunoreaction, lateral flow assay, nanomaterials, point‐of‐care diagnostics, porous materials, signal transduction

## Abstract

Lateral flow assays (LFAs) remain central to point‐of‐care diagnostics because they combine low cost, portability, and operational simplicity. Emerging diagnostic demands, including low‐abundance biomarkers, complex sample matrices, quantitative readout, and multiplexed analysis, increasingly expose limitations that cannot be solved by signal‐label enhancement alone. This Review examines recent LFA advances through a three‐phase framework that couples porous‐material mass transfer, interfacial reaction engineering, and signal transduction. The framework clarifies how membrane and flow design regulate analyte delivery and residence time, how antibody orientation, reaction amplification, and hook‐effect mitigation improve capture efficiency and dynamic range, and how advanced nanolabels, integrated readers, multiplexed formats, and AI/ML‐supported design and analysis expand performance across the assay workflow. By distinguishing intrinsic performance gains from strategies that transfer complexity to reagents, devices, or software, this Review provides design principles for sensitive, quantitative, and translation‐oriented LFA systems.

## Graphical abstract

Lateral flow assays are evolving into interdisciplinary, system‐level sensing platforms. Recent innovations are deconstructed through coupled phases of mass transfer, reaction, and signal transduction, revealing interdependence across porous materials, interfacial chemistry, nanolabels, readout devices, and data‐enabled analysis. The resulting framework maps routes toward integrated, high‐performance, and translation‐ready next‐generation LFAs for emerging diagnostic needs.

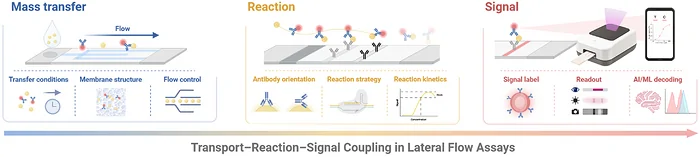

## 1 Introduction

Recent global public health crises, including SARS, Ebola, and COVID‐19, have accelerated interest in shifting part of diagnostic practice from centralized laboratory analysis toward point‐of‐care testing (POCT) [1, 2, 3]. During large‐scale outbreaks, the limitations of conventional laboratory workflows, including restricted throughput, logistical complexity, and delayed turnaround, become particularly apparent. Against this background, lateral flow assays (LFAs) have played an important role in distributed screening and surveillance because they combine rapid response, low cost, operational simplicity, and instrument‐free readout in a readily deployable format [4, 5]. Derived from the underlying principles of paper chromatography and enzyme‐linked immunosorbent assays (ELISA), LFAs have been used since their early commercialization as pregnancy tests in the 1980s [6] and have become among the most widely recognized formats in modern POCT [7, 8, 9]. Their simplicity and deployability make them representative diagnostic platforms aligned with the World Health Organization (WHO) ASSURED criteria (Affordable, Sensitive, Specific, User‐friendly, Rapid and robust, Equipment‐free and Deliverable) [10]. Beyond infectious disease screening, LFAs are now widely used in food safety monitoring [11, 12, 13], including toxin [14] and allergen detection [15], as well as in environmental [16, 17] and clinical analysis [18].

With the continued development of digital health and precision medicine, the expectations placed on POCT platforms have expanded beyond rapid qualitative screening. The evolution from ASSURED to REASSURED criteria [19, 20] placed emphasis on real‐time connectivity, ease of specimen collection, and end‐user accessibility, while also increasing the demand for lower detection limits, improved tolerance to complex sample matrices, and more reliable quantitative or multiplexed analysis. These requirements are particularly evident for low‐abundance biomarkers associated with early‐stage disease, including circulating microRNAs (miRNAs) [21, 22, 23] and exosomes [24], neurodegenerative disease‐related protein fragments such as beta‐amyloids in Alzheimer's disease [25, 26], and trace pathogens present in complex biological or environmental samples [13, 27]. In many of these cases, target concentrations may fall into the femtomolar to attomolar range. As a result, the performance bottlenecks of conventional LFAs are increasingly difficult to explain solely in terms of signal‐label sensitivity. Final assay performance is instead governed by a sequence of coupled processes, including analyte delivery through porous media, molecular recognition and capture at the reaction interface, and the generation, acquisition, and interpretation of the measurable signal.

Recent advances in LFA technology have addressed these bottlenecks from multiple directions. These include advanced optical nanomaterials for signal enhancement [28, 29], biochemical amplification mechanisms such as clustered regularly interspaced short palindromic repeats (CRISPR) and CRISPR‐associated (Cas) systems and isothermal amplification [30], paper‐based microfluidic architectures for improved fluid handling and mass transport [31, 32], and AI/ML approaches that support signal correction, denoizing, interpretation, and data sharing, as well as upstream transport prediction and recognition‐element screening [33, 55, 56]. Existing reviews have summarized these developments from perspectives such as nanolabel type, target class, detection modality, or application scenario. These classifications are useful for mapping progress across the rapidly expanding LFA literature [34, 35, 36, 37, 38, 39]. However, they can also underrepresent the coupled physical and biochemical processes that determine final assay performance. Improvements in label brightness, binding affinity, fluid control, reader design, and computational analysis are not independent; their effects are transmitted through the full assay workflow.

On this basis, this review organizes recent advances in LFAs using a three‐phase framework comprising mass transfer, interfacial reaction, and signal transduction. Rather than treating membranes, recognition chemistry, nanolabels, readers, and algorithms as separable upgrades, we consider them as coupled layers of a porous diagnostic materials system. The mass‐transfer phase describes how analytes and labeled probes are delivered through porous substrates and how membrane engineering, sample pretreatment, buffer composition, and strip geometry regulate residence time and flux. The reaction phase focuses on interface design, including oriented antibody immobilization, reaction amplification, and hook‐effect mitigation strategies that improve capture efficiency, specificity, and usable dynamic range. The signal‐transduction phase examines how formed complexes are converted into readable outputs through optical and nonoptical labels, integrated readout platforms, multiplexed architectures, and AI‐ or machine‐learning‐assisted interpretation. By linking these stages, this Review distinguishes strategies that directly improve intrinsic assay performance from those that shift complexity to reagents, devices, or data processing, and provides a practical framework for rational co‐design of next‐generation POCT platforms.

## 2 Three‐Phase Framework of LFAs

The apparent simplicity of LFAs often conceals a sequence of tightly coupled physicochemical processes. Using a conventional colloidal‐gold colorimetric strip as an example, a typical LFA consists of a sample pad, a conjugate pad, a nitrocellulose (NC) membrane carrying the test line (T‐line) and control line (C‐line), and an absorbent pad arranged in series (Figure 1A) [7, 40]. Once a sample is introduced, liquid migration is driven through the porous strip by capillary action, transporting analytes and labeled probes across multiple functional zones. Although this overall process is often viewed operationally as a single assay step, the formation of a measurable LFA signal is interpreted as progression through three interconnected stages: mass transfer, reaction, and signal transduction (Figure 1B). In the first stage, analytes and probes are delivered through the porous medium; in the second stage, these conjugates undergo molecular recognition and capture at the reaction interface; and in the third stage, the accumulated complexes are converted into an optical, thermal, electrical, or otherwise measurable output. This three‐phase view provides a useful framework for organizing many of the engineering strategies developed to improve LFA performance.

**FIGURE 1:**
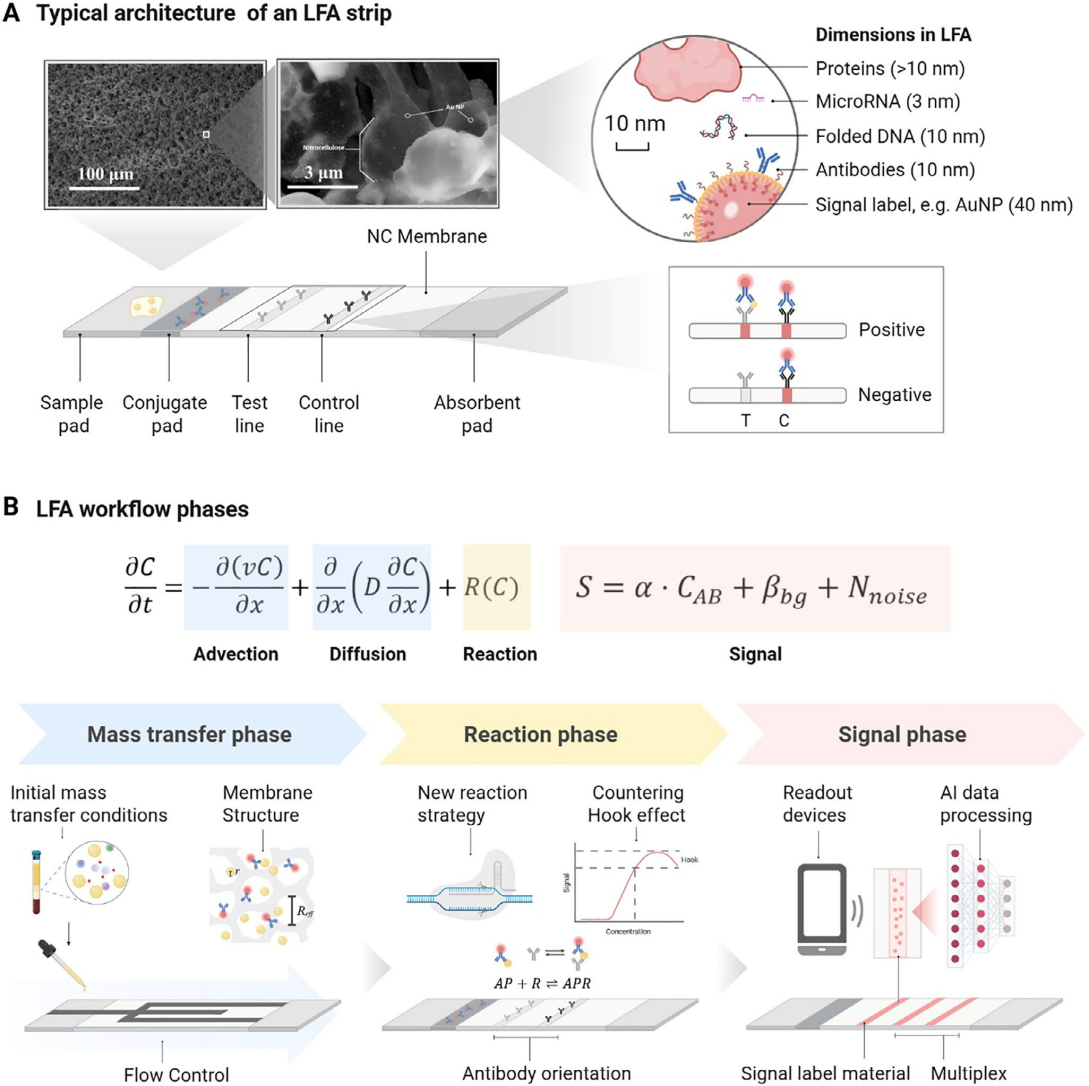
Principles and theoretical framework of LFA. (A) Typical architecture and workflow of an LFA strip, including the sample pad, conjugate pad, nitrocellulose membrane with test and control lines, and absorbent pad. Once the sample is applied, analytes migrate laterally through the porous membrane by capillary action, interact with labeled detection probes, and are captured by immobilized recognition elements at the test line. The inset shows the SEM image of a nitrocellulose membrane. Reproduced with permission from Ref. [41]. Copyright 2017, MDPI. (B) Conceptual deconstruction of LFA signal formation into three sequential and interconnected phases: (i) mass transfer, where analytes are transported via advection and diffusion; (ii) reaction, where analytes bind to immobilized capture probes following reaction kinetics; and (iii) signal transduction, where the quantity of formed complexes is converted into a detectable signal.

From a physical perspective, the macroscopic flow and binding events observed in LFAs arise from coupled transport and interfacial reactions within a porous material. For this reason, the advection–diffusion–reaction equation (ADRE) is widely used for describing analyte transport and consumption before signal formation [39, 42]. In simplified form, the spatiotemporal evolution of analyte concentration in the membrane can be expressed as:

(1) $$\frac{\partial C}{\partial t}=-\frac{\partial \left(uC\right)}{\partial x}+\frac{\partial }{\partial x}\left(D_{eff}\frac{\partial C}{\partial x}\right)+R\left(C\right)$$

where *C* is the analyte concentration in the mobile phase, u is the effective average flow velocity, *D*_eff_ is the effective diffusion coefficient, and *R*(*C*) represents analyte depletion due to interfacial binding in the capture zone. In most LFAs, this sink term is concentrated primarily at the test line, where analytes encounter immobilized receptors under nonequilibrium flow conditions.

Two dimensionless quantities are especially useful for interpreting assay behavior: the Péclet number (*Pe*) and the Damköhler number (*Da*).

(2) $$Pe=\frac{uL}{D_{\mathrm{eff}}}$$

(3) $$\begin{array}{c} Da=\frac{k_{\mathrm{assoc}}R_{0}L}{u}\mathrm{or},\;\mathrm{more}\;\mathrm{generally},\;Da=\frac{\mathrm{reaction}\;\mathrm{timescal}e^{-1}}{\mathrm{transport}\;\mathrm{timescal}e^{-1}} \end{array}$$

where *L* is a characteristic length scale of the capture region, *k*_assoc_ is the association rate constant, and *R*_0_ is the accessible receptor density at the reaction interface. The Péclet number describes the relative importance of advective transport and diffusion, whereas the Damköhler number reflects the competition between reaction kinetics and advective transport. Many conventional LFAs, particularly those operated under rapid capillary flow and low target abundance, tend toward a high‐*Pe* and reaction‐constrained regime. Under such conditions, analytes may be transported efficiently along the strip, but their residence time at the capture zone may be insufficient for binding to approach completion. Reaction efficiency can therefore become a major practical constraint on sensitivity.

**Physical intuition for *Pe* and *Da* in LFAs**

This interpretation provides a useful physical intuition for the design trade‐offs encountered in LFAs. The Péclet number (*Pe*) describes the relative importance of advection and diffusion during analyte transport. In most LFAs, long‐range migration along the strip is predominantly advection‐driven. However, when flow becomes too fast, the residence time of analytes at the test line is shortened, which can reduce the probability of productive capture. The Damköhler number (*Da*) reflects the balance between interfacial reaction kinetics and advective transport. When *Da* ≪ 1, analytes traverse the capture zone faster than binding can occur, and the assay behaves in a reaction‐limited manner. When *Da* ≫ 1, binding is rapid once analytes reach the interface, and transport to the reaction zone becomes the dominant constraint. In practice, many engineering strategies introduced in recent years can be interpreted as attempts to shift LFAs toward a more favorable balance between these competing processes, either by modifying flow fields, increasing residence time, enhancing effective receptor accessibility, or improving signal extraction without directly altering transport–reaction kinetics. The three‐phase framework is not intended to separate these processes rigidly, but rather to provide an analytical structure for understanding where different innovations exert their primary effects.

Recognizing these coupled physical constraints, recent studies have increasingly used ADRE‐based computational models to simulate the full LFA process and guide structural or operational optimization. For example, Schaumburg et al. [43] developed a three‐dimensional numerical model using finite element methods to couple capillary flow, solute transport, and surface reactions (Figure 2A). This model enabled prediction of how complex geometric architectures, such as multipath designs for dengue testing, influence signal distribution and assay uniformity. To better represent practical chromatographic conditions, Zhao et al. [44] coupled ADRE with the Richards’ equation, thereby accounting for nonuniform and time‐dependent capillary flow within the nitrocellulose membrane (Figure 2B). Their model captured spatially and temporally varying flow velocities, including the gradual deceleration of the sample front during membrane penetration. In another example, Xia et al. [45] constructed a multi‐zone kinetic model incorporating both the conjugate pad and membrane, allowing the nonlinear relationship between sample volume, flow velocity, and detection sensitivity to be evaluated. Together, these studies show that computational modelling can help reduce reliance on empirical trial‐and‐error optimization and support more rational assay design.

**FIGURE 2:**
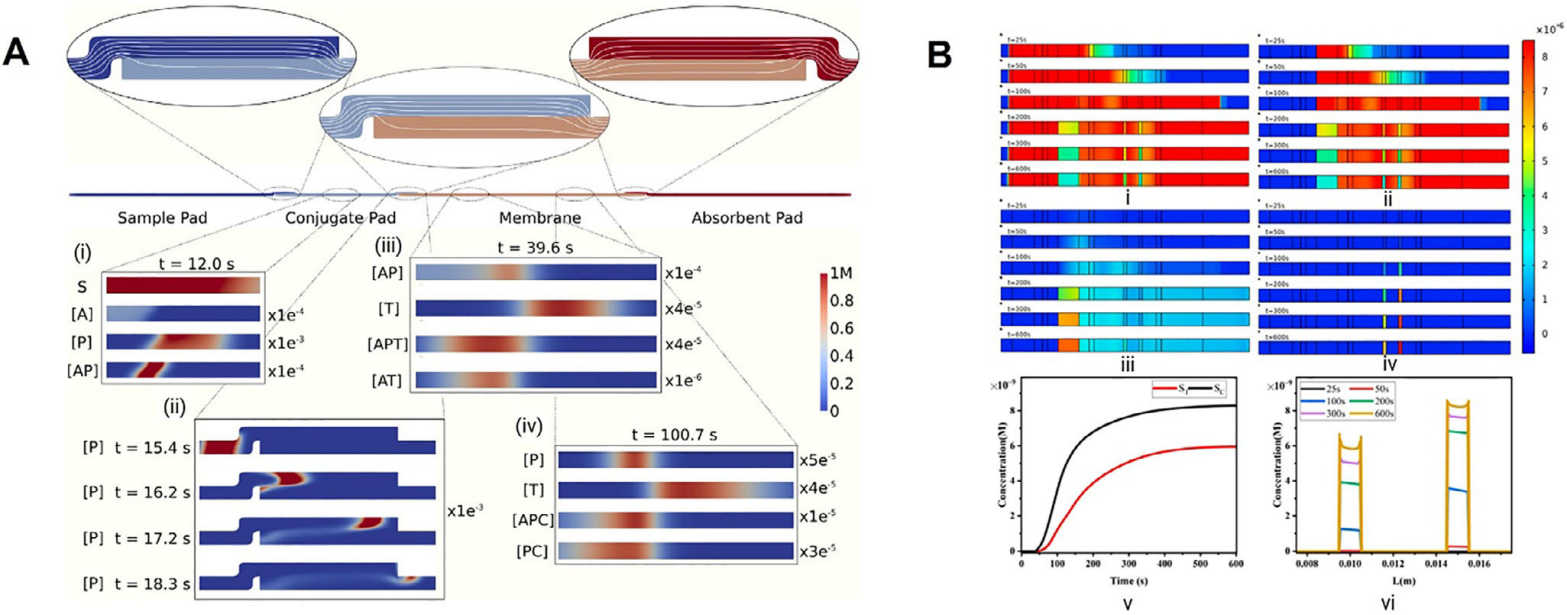
Spatiotemporal fluid dynamics and binding kinetics modelling in LFAs. (A) Evolution of fluid velocity and concentration profiles along a two‐dimensional LFA strip. The computational model tracks coupled mass transfer and reaction processes, highlighting how local velocity profiles and pad‐overlap regions influence particle spreading and sequential immunochemical capture at the test and control lines. Adapted with permission from Ref. [43]. Copyright 2018, Elsevier. (B) Simulation of a sandwich‐format LFA reaction process. The model maps the spatial distribution of analytes, reporter probes, and conjugated complexes during chromatography, enabling quantitative analysis of transient signal generation and localized diffusion effects within porous capture zones. Adapted with permission from Ref. [44]. Copyright 2024, MDPI.

Viewed in this way, the major performance determinants of each stage can be summarized more systematically (Table 1). In Phase I, mass transfer determines how efficiently analytes and labeled probes are delivered to the reaction zone. Key variables include the initial analyte concentration (*C*_0_), flow velocity (*u*), effective diffusion coefficient (*D*_eff_), residence time (*t*_res_), and *Pe*. Strategies such as buffer formulation, nanoprobe size, membrane modification, geometric constriction, and external flow regulation mainly act by changing analyte availability, wetting behavior, and local transport dynamics before capture occurs. In Phase II, reaction‐stage performance is governed by association and dissociation rate constants (*k*_assoc_ and *k*_disassoc_), the accessible receptor density (*R*_0_), and the resulting reaction–transport balance expressed by *Da*. This stage includes strategies such as oriented antibody immobilization, reaction amplification, and hook‐effect mitigation. Once specific complexes are formed, the emphasis shifts in Phase III (signal transduction) to the conversion of molecular binding into a readable output. At this stage, key considerations include the signal transduction coefficient, background interference, stochastic noise, and the accuracy of subsequent signal interpretation. Advances in signal labels, reader architecture, and AI‐assisted analysis all contribute to final assay performance, particularly in low‐signal or high‐background settings.

**TABLE 1:** Conceptual summary of key processes, parameters, bottlenecks, engineering strategies, and translational concerns in the three‐phase LFA framework.

Phase	Dominant process	Key parameters	Typical bottlenecks	Representative engineering strategies	Translational concern
Phase I: Mass transfer	Delivery of analytes and labeled probes through the porous strip	*C*_0_, *u*, *D*_eff_, *Pe*, *t*_res_, wetting behavior	Rapid capillary flow; insufficient residence time; poor wetting; matrix fouling; nonspecific retention	Buffer formulation; nanoprobe size engineering; membrane modification; pore‐structure tuning; geometric constriction; assisted flow control; microfluidic mixing	Compatibility with roll‐to‐roll manufacturing; membrane batch variability; robustness in complex matrices
Phase II: Reaction	Molecular recognition, capture, and complex formation at the T‐line	*k*_assoc_, *k*_disassoc_, *R*_0_, receptor accessibility, *Da*, reaction time	Low capture efficiency; random antibody orientation; steric hindrance; weak association under flow; dissociation; hook effect; biochemical cross‐reactivity	Oriented immobilization; bioaffinity anchoring; photochemical immobilization; recombinant fusion proteins; CRISPR‐Cas‐mediated reaction amplification; temporal flow delay; kinetic monitoring	Reagent stability; retention of antibody activity; integration of amplification steps; preservation of simple workflow
Phase III: Signal	Conversion of captured complexes into readable optical, thermal, electrical, magnetic, spectroscopic, or digital outputs	α, *R* ^2^, stochastic noise (*N_noise_*), background interference (β_*bg*_), classification accuracy	Spectral overlap, color‐channel crosstalk, and decoding ambiguity in multiplexed assays.	Colorimetric, fluorescent, UCNP, SERS, photothermal, electrochemical, nanozyme, and multimodal labels; integrated readers; wearable interfaces; AI/ML‐assisted processing	Hardware burden; cost; calibration transferability; cross‐device robustness; regulatory acceptance of AI‐assisted interpretation

The ADRE framework is most directly relevant to Phase I and Phase II, where transport and interfacial reaction are explicitly coupled. Phase III is governed more strongly by the physical principles associated with signal generation and detection, including optical, electrical, thermal, or spectroscopic transduction. In addition to the signal transduction coefficient (α), this stage is also shaped by background interference, stochastic noise, classification accuracy, and the quantitative fidelity of downstream readout models, often expressed through metrics such as accuracy and the coefficient of determination (*R* ^2^). At the materials level, advanced signal probes, including upconversion nanoparticles (UCNPs) [46, 47], gold nanoparticles (AuNPs) [28, 48], surface‐enhanced Raman scattering (SERS) tags [49], photothermal materials [50, 51], and nanozymes [52, 53], have substantially expanded the detectable range of LFAs. Even so, treating the assay as a continuous sequence from analyte delivery to signal interpretation remains useful because it helps place recent developments in membrane engineering, reaction amplification, portable instrumentation, and computational/AI analysis within a single workflow‐based perspective.

The three‐phase framework provides a clear way to organize the key processes in lateral flow assays (LFAs). In practice, transport, target recognition, and signal generation form a connected sequence that links the three phases. Design choices introduced at any stage can propagate through the workflow and influence performance in the others. For each design factor, it is therefore useful to distinguish its primary phase of action from secondary effects that propagate into the other stages of the assay. Attention to these connections is therefore important for interpreting assay behavior and avoiding the isolated optimization of individual components.

Nanoparticle labels provide a direct link between transport, recognition, and readout. During Phase I, their size and surface properties influence diffusion and movement through the porous membrane. At the test line, the same properties shape antibody loading, multivalent interactions, and steric accessibility. Once labels accumulate, they determine the optical or photothermal contrast available for readout. This coupled role is reflected in AuNP‐based LFA studies that relate nanoparticle design to both receptor interactions and visual or thermal signal generation [63].

Membrane architecture establishes the physical setting in which transport and capture take place. Pore structure, thickness, and wettability set the flow rate and analyte residence time during Phase I. They also shape reagent distribution, protein adsorption, and the accessibility of immobilized capture elements at the reaction interface. Differences in wetting behavior and local label accumulation can then appear as changes in background and readout reproducibility. The coupled flow‐reaction analysis reported by Jia et al. captures this connection between membrane structure, transport, and target capture [54].

AI/ML enters this framework in a different way. Its most established role in LFA remains Phase III, where it supports image‐based quantification, classification, calibration, and multiplex decoding. Its value can nevertheless extend upstream. Artificial neural networks have been used to predict NC membrane wicking behavior from formulation and manufacturing data, informing material selection before strip fabrication [55]. Machine‐learning approaches for antibody–antigen affinity prediction likewise offer a route for prioritizing recognition elements before experimental screening [56]. Together, these applications position AI/ML as a design‐enabling tool across the LFA workflow, while Phase III remains its most mature application.

The following sections discuss representative factors according to the phase in which they are most directly examined. Where relevant, their consequences for the other phases are also considered, thereby connecting component‐level optimization with overall assay performance.

## 3 Phase I: Mass Transfer

Mass transfer represents the initial stage of assay operation, during which analytes and labeled probes are transported through the porous strip toward the reaction zone. The central objective of this stage is to deliver analytes to the capture interface with sufficient flux within an operationally useful assay time. In this sense, mass transfer does not simply determine whether the analyte reaches the test line; it also shapes the local microfluidic environment in which subsequent binding takes place. In this way, transport behavior is closely linked to the ultimate sensitivity, reproducibility, and practical operating window of the assay [57]. In many conventional LFAs, especially under low‐abundance conditions, incomplete analyte delivery or insufficient interaction time can become an important upstream limitation, even before issues of molecular recognition or signal generation arise.

Most LFAs rely primarily on passive capillary flow through porous materials, and the chromatographic migration of liquid can be described, in simplified form, by the Lucas–Washburn relationship [58]:

(4) $$L^{2}=\frac{\gamma r_{eff}cos\theta t}{2\eta }$$

where γ is the liquid surface tension, *r_eff_* is the effective pore radius, θ is the contact angle, and η is the fluid viscosity. The standard wetting‐front form shows that $L^{2}$ is proportional to *t*. Accordingly, the instantaneous front velocity decreases as the wetting front advances and with time; it should not be interpreted as a constant chromatographic velocity. In practical NC membranes, γ, *r_eff_*, and θ are effective parameters that represent a heterogeneous pore network. This relationship highlights that transport behavior in LFAs is governed jointly by material structure and liquid properties. From this perspective, optimization of Phase I generally follows two broad directions. One is to slow or redistribute convective transport to extend analyte residence time at or near the test line, thereby increasing the opportunity for productive capture in the subsequent reaction stage. The other is to improve wettability and transport uniformity by regulating the physicochemical environment of the flowing sample, which helps reduce local heterogeneity, suppress nonspecific retention, and stabilize assay performance across the membrane.

Recent work can therefore be understood as an effort to regulate mass transfer across multiple scales. At the level of the mobile phase, transport can be influenced from the outset by the size of nanolabels and the composition of the running buffer, both of which alter effective diffusivity, wettability, and flow stability. At the level of the porous substrate, membrane modification or replacement can reshape pore architecture, fluid–surface interactions, and local transport resistance. At the device level, geometric redesign and external actuation can further redistribute flow fields and interaction times along the strip.

### 3.1 Initial Mass Transfer Conditions: Sample Matrix, Particle Size, and Buffer

At the beginning of Phase I, the analyte, antibody‐conjugated signal probes, and sample matrix together define the initial transport environment of the assay. In simplified laboratory systems, this environment is often represented by a controlled running buffer. In practical LFAs, however, the applied sample may be whole blood, plasma, serum, saliva, urine, respiratory swab extract, food extract, wastewater, soil leachate, or plant‐derived fluid. These matrices differ markedly in viscosity, particulate load, pH, ionic strength, endogenous background, and fouling potential, thereby influencing capillary migration, probe release, nonspecific retention, and downstream binding. For this reason, Phase I optimization should consider not only nanoprobe size and buffer composition, but also the compatibility of the sample matrix and any pretreatment steps integrated into the strip or device.

#### 3.1.1 Sample Type and Integrated Pretreatment

Before particle size and buffer composition are considered, the sample matrix itself defines the initial mass‐transfer environment of an LFA. In practical applications, LFAs are rarely exposed only to simplified running buffers; instead, they must operate in diverse clinical, environmental, agricultural, and food‐related matrices, including whole blood, plasma, serum, saliva, respiratory swab extracts, urine, stool extracts, food extracts, milk, wastewater, soil leachates, and plant‐derived fluids. These matrices differ substantially in viscosity, particulate content, endogenous background, pH, ionic strength, inhibitor load, and target stability, thereby influencing capillary migration, probe release, nonspecific retention, analyte availability, and signal background. As summarized in Table 2, sample type is therefore not merely an application variable, but a first‐layer Phase I design parameter that should be considered together with membrane properties, buffer formulation, probe design, and downstream signal readout.

**TABLE 2:** Matrix‐dependent Phase I constraints and integrated pretreatment strategies in LFAs.

Sample matrix	Typical applications	Key Phase I challenges	Pretreatment strategies	Commercial maturity
Whole blood	CRP, cardiac markers, infection serology	Cells, viscosity, hemoglobin, clotting	Plasma separation pad; depth filtration; anticoagulant buffer	Mature/commercial
Plasma/serum	Inflammation, cardiac, cancer biomarkers	Protein fouling, matrix background	Blocking buffer; dilution; antifouling membrane	Mature in clinical POCT
Saliva	Respiratory infection, hormones, oral biomarkers	Mucins, variable viscosity, low target level	Mucolytic buffer; filtration; volume control	Commercial/developing
Nasal or throat swab	SARS‐CoV‐2, influenza, bacterial infection	Sampling variability, mucus, extraction dependence	Extraction buffer; lysis pad; swab elution	Commercial
Urine	Pregnancy, ovulation, drugs, kidney markers	Dilution variability, salts, pH	Buffering pad; normalization; enrichment membrane	Highly commercialized
Stool extract	GI infection, occult blood, inflammation	Particulates, pigments, enzymes	Extraction tube; dilution; filtration cap	Commercial/challenging
Food extract	Allergens, toxins, pathogens, residues	Fat, pigments, particles, solvents	Homogenization; filtration; immunoenrichment	Commercial/application‐specific
Water/wastewater	Pathogens, toxins, surveillance	Low target level, inhibitors, large volume	Filtration; concentration; paper purification	Research/field demonstration
Soil leachate	Pesticides, heavy metals, plant pathogens	Humic substances, particulates, pH	Sedimentation; filtration; extraction buffer	Mostly research
Plant extract	Plant viruses, fungi, mycotoxins	Pigments, phenolics, debris	Grinding bag; dilution; filtration	Commercial/developing
Veterinary samples	Zoonoses, mastitis, hormones	Matrix‐dependent interference	Filtration; dilution; sample‐specific pads	Commercial/developing

Among these matrices, whole blood is one of the most clinically important and technically challenging samples for LFA‐based POCT. Although finger‐prick whole‐blood testing is attractive for decentralized diagnostics, red blood cells, clotting, hemoglobin coloration, and high viscosity can obstruct membrane flow and interfere with visual or optical readout. Commercial LFA devices therefore often incorporate plasma‐separation materials, typically based on glass–fiber or cellulosic depth filters, which retain blood cells while allowing plasma to migrate into the downstream strip. Such components are already compatible with scalable LFA manufacturing and can reduce the need for centrifugation. More integrated sample‐to‐answer approaches have also been explored. For example, self‐pressure‐driven plasma‐separation devices can extract plasma from microliter‐scale whole‐blood samples without electrical equipment and deliver it directly to rapid diagnostic strips [59]. In nucleic‐acid testing, pretreatment may extend beyond filtration to include lysis, extraction, amplification, and controlled transfer to the LFA readout zone, as illustrated by paper microfluidic reverse‐transcription loop‐mediated isothermal amplification (RT‐LAMP) systems that integrate amplification with lateral‐flow detection [60].

Other matrices impose distinct Phase I constraints. Saliva, respiratory swabs and urine enable noninvasive testing, but viscosity, mucins, salts, pH and dilution vary with collection and sampling time. Stool, food and milk extracts add particulate, enzymatic, lipid or protein backgrounds, whereas wastewater, soil leachates and plant extracts may contain solids, humic substances, pigments, microbial debris, metabolites and buffer residues. Such matrices often require dilution, filtration, homogenization, defatting, concentration, inhibitor removal, or paper‐based purification before reproducible lateral‐flow transport can occur [61]. Pretreatment therefore does more than remove visible debris: it conditions the target, labeled probe, and membrane interface for stable mass transfer and recognition.

Translational maturity remains uneven. Plasma‐separation pads, extraction buffers, and treated sample pads are already compatible with standard LFA formats, whereas self‐powered separators, microfluidic filters, paper‐based nucleic‐acid extraction, and environmental processing modules are mostly at research or early‐translation stages. Their value depends on improving matrix tolerance and sensitivity without sacrificing low cost, short assay time, few user steps, storage stability, or manufacturability. Integrated pretreatment is therefore best viewed as upstream mass‐transfer engineering. For next‐generation POCT, the most practical designs will process samples only as much as needed for reliable transport, nanoprobe migration, and interfacial capture, while preserving the simplicity that defines LFA testing.

#### 3.1.2 Effect of Particle Size

For nanoparticle‐based nanolabels, particle size is an important design variable because it affects not only the final signal readout, but also the transport behavior of probes before capture. In conventional LFAs, labels such as colloidal AuNPs ultimately serve as optical or luminescent reporters in Phase III [48], but they must first be efficiently delivered to the test line through the porous structure of the strip. According to the Stokes–Einstein relation [39, 62], the effective diffusion coefficient of a nanoparticle is inversely related to its hydrodynamic radius. As particle size increases, diffusivity (*D_eff_*) decreases, which in turn elevates the effective Péclet number (*Pe*) of the system (Figure 3A). Under such conditions, particles become more strongly coupled to the advective flow and less able to diffuse laterally toward pore walls or capture interfaces. In transport terms, this can reduce the probability of productive encounters with immobilized receptors. At the same time, however, larger particles may provide clear advantages at later stages of the assay, including higher molar extinction coefficients, greater luminescence output, and larger surface areas for antibody loading, all of which can improve multivalent binding and enhance signal generation. Particle size is therefore associated with a characteristic trade‐off between transport efficiency and signal or binding capacity in LFA design.

**FIGURE 3:**
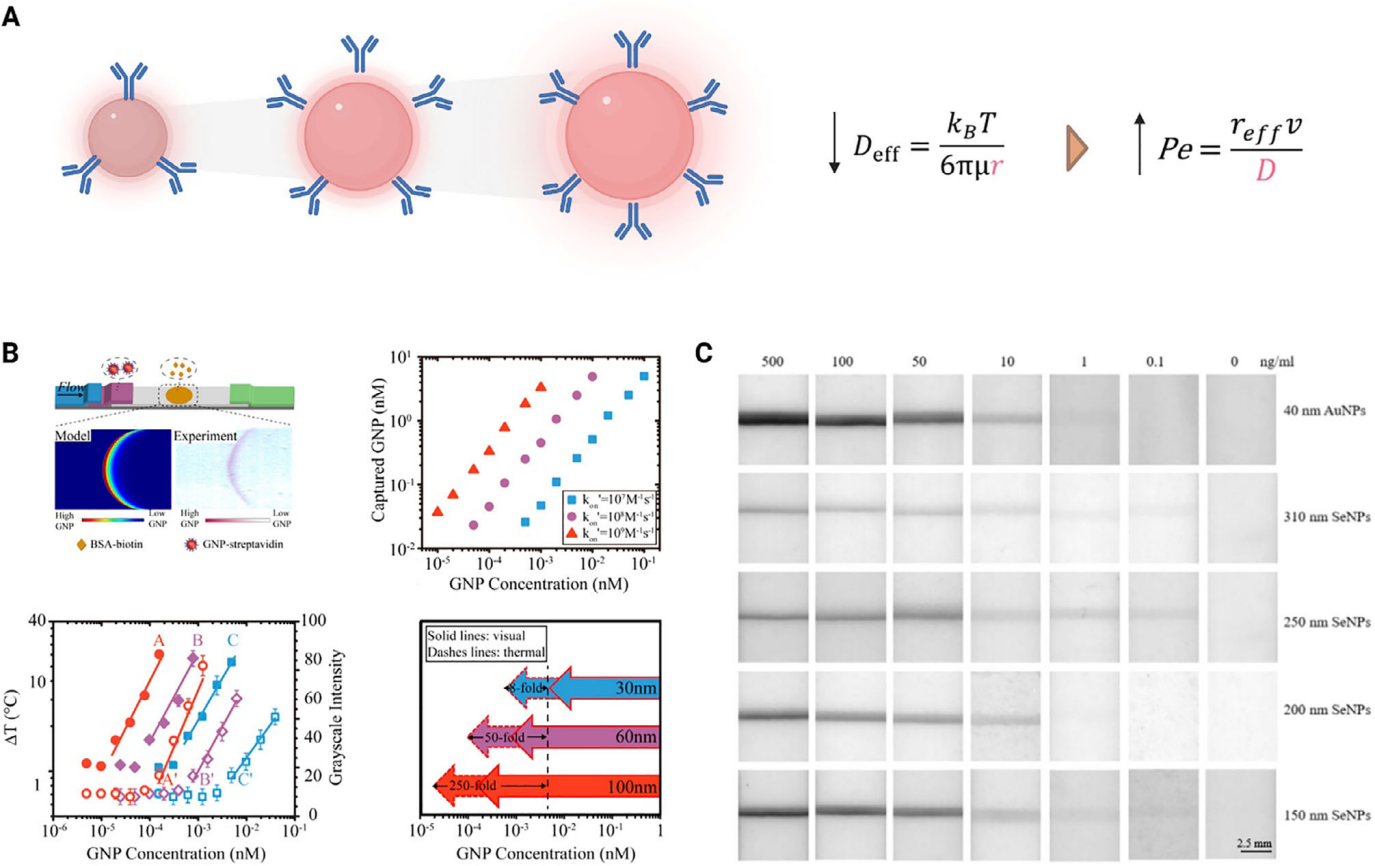
Size‐dependent effects of nanoparticle probes on mass transfer and signal output in LFAs. (A) Schematic illustration of particle‐size‐dependent trade‐offs in LFAs. Larger nanoparticles generally provide higher molar extinction coefficients and greater antibody‐loading capacity, which can enhance multivalent binding and increase signal output in Phases II and III. However, increasing particle size also reduces the effective diffusion coefficient and increases the Péclet number (Pe) during Phase I. Under high‐Pe conditions, probes become more strongly advected by the main flow and less able to diffuse transversely toward capture sites, reducing the probability of productive collision with immobilized antibodies. (B) Effect of AuNP size (30, 60, and 100 nm) on LFA performance. Combined computational modelling and experimental thermal/visual readouts showed that the integrated 100 nm platform balanced the transport penalty of reduced diffusion against enhanced antibody loading, multivalent binding, and photothermal output. The performance gain should not be interpreted as a particle‐size effect alone. Adapted with permission from Ref. [63]. Copyright 2017, ACS. (C) Comparison of test‐line visibility using conventional 40 nm AuNPs and larger selenium nanoparticles (SeNPs, 150–310 nm) for interleukin‐6 (IL‐6) detection. The result illustrates that excessively large probes may suffer from steric hindrance and impaired transport through the porous membrane, despite improved optical contrast. Adapted with permission from Ref. [64]. Copyright 2023, ACS.

This balance has been examined in detail for AuNP‐based LFAs. Zhan et al. [63] combined full‐process computational modelling with experimental validation to compare 30, 60, and 100 nm AuNPs in an integrated thermal‐contrast amplified LFA platform (Figure 3B). In this platform, the 100 nm AuNP format provided a favorable balance between acceptable transport through the strip, antibody loading capacity, multivalent binding, and photothermal response. Relative to the 30 nm reference format, the integrated 100 nm platform improved the sensitivity of C‐reactive protein detection in clinical samples by 256‐fold and expanded the quantitative linear dynamic range from 3 orders of magnitude to 6 orders of magnitude while maintaining stable transmembrane flow. This improvement should not be attributed to particle size alone, because antibody loading capacity, multivalent binding, photothermal readout, and the broader assay design also contributed to the observed performance. The result instead illustrates that the optimum probe design is the one that balances diffusional mobility, interfacial capture, and signal amplification within a given assay architecture.

A similar pattern is observed for other nanoparticle systems. Bradley et al. [64] investigated selenium nanoparticles (SeNPs) with diameters of 150–310 nm for interleukin‐6 (IL‐6) detection and compared them with conventional 40 nm AuNP probes (Figure 3C). The larger SeNPs benefited from improved optical response and increased payload capacity, allowing the limit of detection (LOD) to be reduced from 1 to 0.1 ng mL^−^^1^. This gain was accompanied, however, by a clear transport penalty: excessively large probes experienced steric hindrance and were more readily trapped within the nitrocellulose matrix, which reduced effective mass transfer to the test line and weakened the visible signal intensity. This example further illustrates that particle size in LFAs should not be treated solely as a signal parameter. Rather, it is an upstream transport variable that simultaneously influences diffusion, collision probability, interfacial loading, and eventual readout strength. As such, particle‐size engineering should be considered as part of a coupled transport–reaction–signal optimization problem, in which colloidal stability must also be controlled through appropriate surface chemistry, blocking proteins, surfactants, ionic strength, and pH to avoid aggregation during migration.

#### 3.1.3 Effect of Buffer

If particle size defines one part of the initial transport environment, the running buffer defines the other. In LFAs, the buffer is not merely a passive carrier for analytes and labeled probes; it regulates the physicochemical environment in which capillary migration, probe stability, nonspecific adsorption, and subsequent recognition occur. Its composition influences surface tension, viscosity, ionic strength, pH, wetting behavior, and probe dispersion, all of which affect the effective transport parameters described in Phase I. These effects become particularly important when LFAs are applied to complex matrices such as serum, saliva, whole blood, or environmental samples, where aggregation, fouling, unstable flow fronts, and matrix‐induced background can compromise reproducibility [65, 66]. Buffer optimization is therefore a direct and practical way to stabilize mass transfer before the reaction and signal stages are reached.

Several classes of additives are commonly used to tune these properties (Table 3). Surfactants primarily regulate wetting and flow uniformity by reducing surface tension and improving liquid penetration into the nitrocellulose membrane. Nonionic surfactants, such as Tween‐20, Triton X‐100, and Brij 35, are widely used because they improve membrane wettability while remaining relatively mild toward proteins and antibodies [67]. Ionic surfactants, including SDS, CHAPS, and CTAB, can provide stronger solubilization or cell‐disruption effects, but must be used cautiously because excessive concentrations may denature proteins or perturb antigen–antibody interactions. In Phase I terms, surfactants help stabilize the advancing fluid front, reduce local flow heterogeneity, and limit nonspecific retention of probes within the porous matrix.

**TABLE 3:** Functional roles and practical considerations of common additives in LFA running buffers.

Category	Representative agents	Main roles in Phase I	Practical considerations
Nonionic surfactants	Tween‐20, Triton X‐100, Brij 35	Reduce surface tension; improve wettability; stabilize the fluid front; reduce nonspecific retention	Broadly compatible with proteins; commonly used in standard LFA buffers
Ionic surfactants	SDS, CHAPS, CTAB	Promote solubilization; assist cell or membrane disruption; release encapsulated targets	Useful for difficult matrices but may denature proteins or disrupt binding if overused
Protein blockers	BSA, casein, skim milk	Suppress nonspecific adsorption; reduce background; stabilize probes; limit aggregation	Choice depends on matrix complexity, background level, and probe/antibody compatibility
Salts and buffers	PBS, HEPES, Tris, NaCl	Maintain pH and ionic strength; regulate electrostatic interactions; improve transport and binding stability	Excess ionic strength may alter antibody binding or promote probe aggregation
Polymers and sugars	PEG, PVA, sucrose, trehalose	Tune viscosity; slow capillary flow; protect dried probes; support controlled rehydration	Can improve residence time and probe release, but excessive viscosity may delay assay completion
Preservatives	Sodium azide, ProClin series	Maintain reagent stability; inhibit microbial growth	Limited direct effect on transport; concentration must remain compatible with biomolecules

Protein blockers, such as bovine serum albumin (BSA), casein, and skim milk, mainly reduce nonspecific adsorption between labeled probes and the membrane. By occupying exposed hydrophobic or charged sites on nitrocellulose, they suppress background signal and help preserve probe mobility during lateral transport. They can also stabilize nanoparticles in suspension and reduce aggregation, which is particularly important for larger or more surface‐reactive probes. Salts and buffering agents, such as PBS, HEPES, Tris, and NaCl, maintain pH and ionic strength [68], thereby modulating electrostatic interactions among analytes, probes, capture antibodies, and the membrane. These effects influence both transport stability in Phase I and binding efficiency in Phase II.

Polymers and sugars provide another level of transport control. Polymers such as PEG and PVA can increase viscosity and slow capillary migration, thereby extending residence time near the reaction zone. Sugars such as sucrose and trehalose are commonly used to protect dried probes and promote controlled rehydration when the sample enters the strip. This rehydration process is important because incomplete or uneven probe release from the conjugate pad can distort downstream transport and reduce assay reproducibility. Preservatives such as sodium azide and ProClin have limited direct influence on mass transfer, but they are important for maintaining long‐term reagent stability in practical LFA devices.

In practice, buffer design usually requires a composite formulation rather than a single additive. Surfactants, blockers, salts, polymers, sugars, and preservatives are combined to balance membrane wetting, flow velocity, probe dispersion, nonspecific adsorption, and reagent stability. From the Phase I perspective, the running buffer should therefore be regarded as an engineered transport medium that defines the initial microenvironment of the assay. A well‐optimized buffer can improve matrix compatibility, maintain a stable fluid front, reduce probe aggregation, and create more reproducible conditions for subsequent capture and signal generation.

### 3.2 Redesigning the Membrane Matrix

In LFAs, the membrane matrix forms the core physical scaffold of the mass‐transfer network. In conventional strips, the nitrocellulose (NC) membrane is the principal substrate through which liquid migration, analyte delivery, and interfacial capture are coordinated [31]. Its structural properties, including effective pore size, porosity, and tortuosity, together with its surface chemistry, influence key parameters in capillary transport, particularly the effective pore radius and solid–liquid contact angle described in the Lucas–Washburn framework. Membrane engineering has therefore become an important route for regulating Phase I behavior. By modifying surface physicochemistry, reshaping internal pore architecture, or integrating alternative substrate materials, it becomes possible to influence local wetting, capillary velocity, analyte residence time, and nonspecific retention in ways that are difficult to achieve through buffer composition or probe design alone. In some cases, membrane redesign also affects the local reaction environment by changing receptor accessibility, reagent release, or optical background, thereby linking Phase I regulation with downstream reaction and signal recovery.

#### 3.2.1 Modification and Functionalization of Membrane Matrices

Commercial NC membranes remain the standard in most LFA devices because they combine strong capillary wicking with high protein‐binding capacity, allowing aqueous samples to traverse the strip rapidly while preserving the operational simplicity expected of POCT formats. At the same time, this rapid transport can shorten the effective interaction time available for target capture. Much of the work on membrane modification has therefore aimed not to replace NC outright, but to preserve its practical advantages while locally slowing, redistributing, or stabilizing transport so that analytes and labeled probes encounter the reaction interface more efficiently.

A useful mechanistic basis for this design logic has been provided by advection–diffusion–reaction analysis of NC membranes [42]. This study showed that LFA performance is not governed simply by whether capillary flow is faster or slower, but by how pore architecture couples longitudinal advection with through‐thickness diffusion and interfacial reaction. In particular, larger pores in the lateral direction can maintain relatively rapid bulk transport along the strip, whereas smaller pores in the thickness direction can promote diffusion toward the capture interface and improve immunoreaction efficiency. This structure–function relationship suggests that NC membranes should be viewed not only as passive wicking substrates, but as three‐dimensional transport–reaction matrices whose internal morphology can be rationally optimized to balance assay speed and sensitivity.

A complementary flow‐reaction model by Jia et al. showed that pore‐size variation alters the balance among capillary transport, diffusion, reactive surface area, and target capture. Their heterogeneous NC design, with a smaller‐pore test‐line region and larger pores elsewhere, illustrates how membrane structure can improve sensitivity without simply slowing the entire strip [54].

Alongside physics‐based transport models, data‐driven prediction can provide a complementary route for membrane design. Dissanayake et al. used an artificial neural network to predict the wicking rate of NC membranes from formulation and manufacturing data [55]. This example shows that AI/ML can support Phase I before strip fabrication by helping identify membrane compositions and process conditions that are compatible with the desired transport behavior. Such predictive tools do not replace mechanistic analysis, but can make the exploration of membrane‐design space more efficient.

Several experimental studies illustrate this principle at the level of membrane morphology and surface functionalization. Local mechanical compression of NC membranes can reduce the effective pore radius near the reaction region, slow capillary flow, and prolong analyte residence time, leading to improved CRP sensitivity [69]. Incorporating cellulose nanofibers (CNFs) into NC membranes provides a complementary strategy: the CNF network alters pore topology, increases specific surface area, and retards capillary transport, producing a reported 20‐fold sensitivity enhancement in a nucleic‐acid LFA for *Staphylococcus aureus* [70]. Chemical functionalization can further improve the local capture environment. In a fluorescent LFA for the traumatic brain injury biomarker ubiquitin carboxy‐terminal hydrolase L1 (UCH‐L1), the introduction of a trace amount of graphene oxide (GO) into the detection region created a GO‐enriched capture layer with abundant oxygen‐containing functional groups for antibody immobilization [71]. Relative to the unmodified NC membrane, this GO‐coated zone increased fluorescence intensity by 2–3‐fold and reduced the limit of detection to 11.1 pg mL^−^^1^. These examples show that NC modification can improve Phase I performance not only by changing flow velocity, but also by enhancing the physicochemical quality and accessibility of the local reaction interface.

#### 3.2.2 Replacement and Integration of Novel Membrane Substrates

Although NC remains the benchmark in most commercial formats, its limitations become more apparent in complex or highly fouling sample matrices. Its relatively hydrophobic character can promote nonspecific adsorption when exposed to viscous, protein‐rich, or lipid‐rich samples, such as whole blood, mucus, or untreated environmental suspensions. These interactions can hinder migration of low‐abundance targets, increase background, and reduce reproducibility. Alternative substrate materials and integrated membrane architectures have therefore been explored to improve antifouling behavior, wetting control, reagent handling, and background suppression.

One important direction is the construction of multilayer or three‐dimensional paper‐based architectures. Rather than relying on a single homogeneous membrane, these systems combine layers with different wetting characteristics and reagent functions, allowing vertical and lateral flow paths to be coordinated within one device. In a three‐dimensional nucleic‐acid lateral flow assay (NALFA), Au deposition and Ag staining reagents were predried in stacked paper layers with defined hydrophobic and hydrophilic regions, enabling sequential liquid transport and one‐step dual‐metal signal amplification after sample loading (Figure 4A) [72]. This architecture spatially isolated potentially interfering amplification chemistries and autonomously coordinated their rehydration and migration, retaining a simple one‐step workflow while enabling SARS‐CoV‐2 detection within 25 min and achieving a reported LOD of 2.24 nM.

**FIGURE 4:**
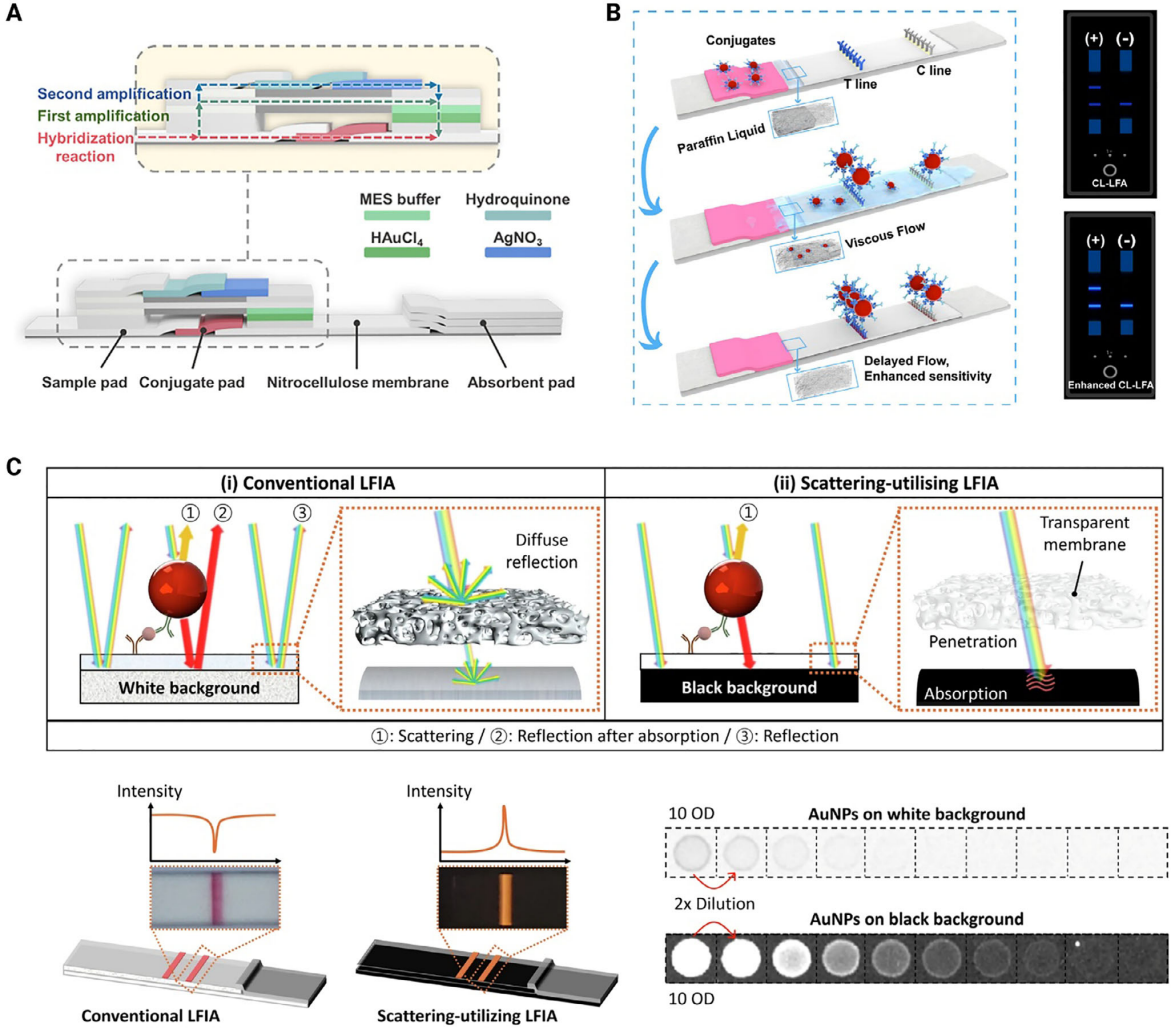
Membrane‐matrix engineering for regulating mass transfer, reagent handling and signal recovery in LFAs. (A) Three‐dimensional paper‐based NALFA architecture with stacked hydrophobic and hydrophilic layers enables spatial reagent separation, sequential liquid transport, and one‐step dual‐metal signal amplification. Adapted with permission from Ref. [72]. Copyright 2025, ACS. (B) Liquid‐phase barrier modification of NC membranes increases local viscosity and prolongs analyte residence time near the test and control lines, thereby improving chemiluminescent LFA signal generation. Adapted with permission from Ref. [74]. Copyright 2025, ACS. (C) Transparentized NC membranes combined with an absorbing backing layer suppress diffuse optical reflection and enhance plasmonic scattering signals from AuNP labels, linking membrane engineering with downstream signal recovery. Adapted with permission from Ref. [75]. Copyright 2025, Springer Nature.

Temporal regulation of sample flow provides another route to improving reaction efficiency. Dissolvable barriers based on polyvinyl alcohol (PVA), together with dense cellulose fibers, have been used to delay sample progression and lengthen premixing between target analytes and recognition probes, yielding an overall 20‐fold enhancement in assay sensitivity [73]. More recent liquid‐phase barrier modification extends this logic directly into the NC membrane by introducing localized high‐viscosity regions around the test and control lines (Figure 4B) [74]. These barriers slow the passage of analytes and labeled probes through the capture zones, prolonging the effective residence time available for complex formation. In this sense, membrane design becomes a form of passive fluidic programming rather than merely a choice of substrate.

Membrane redesign can also influence analytical performance by reducing the optical background against which weak signals are detected. In a plasmonic‐scattering LFA, transparentization of the NC membrane combined with an absorbing backing layer suppressed diffuse reflection from the membrane matrix and enhanced the visibility of AuNP scattering signals (Figure 4C) [75]. Although this strategy is closely connected to the signal‐readout stage discussed later, it reinforces the broader point that the membrane matrix affects not only upstream mass transfer and reaction kinetics, but also downstream signal recovery.

Overall, membrane redesign provides a complementary route to sensitivity improvement alongside signal‐label engineering. Rather than acting only as a passive wicking scaffold, the membrane can be engineered to regulate lateral advection, through‐thickness diffusion, interfacial reaction efficiency, nonspecific adsorption, reagent release, and optical background. Whether implemented through local compression, nanoscale compositing, surface functionalization, multilayer paper architectures, dissolvable or liquid‐phase barriers, or optical‐background reduction, membrane engineering shapes the transport–reaction–signal microenvironment before final readout is obtained. However, it remains less commercially mature than label engineering, largely because small variations in pore architecture, surface chemistry, reagent loading, optical background, and membrane batch properties can strongly affect flow reproducibility and assay robustness. Future membrane redesign will therefore need to balance improved mass‐transfer control with manufacturability, batch consistency, and compatibility with standard strip production.

### 3.3 Modifying Strip Geometry

In addition to regulating buffer composition or redesigning the porous membrane itself, Phase I can also be engineered at the device level by modifying strip geometry and the associated flow field. Compared with membrane modification, this approach operates at a more macroscopic scale: rather than directly altering pore chemistry or substrate composition, it reshapes the spatial distribution of flow resistance, residence time, sample utilization, and local analyte concentration along the strip. Importantly, geometry‐based regulation should not be viewed simply as a way to slow capillary flow. Instead, it provides a demand‐oriented design layer. Assays targeting low‐abundance biomarkers may benefit from local enrichment and prolonged interaction time, whereas emergency or time‐critical assays may require rapid, directional transport with minimal residual background.

For biomarkers that require stronger analytical signals, one direct strategy is to concentrate the sample stream near the capture zone. The “narrow‐gate” architecture reported by Jin and co‐workers illustrates how a simple change in strip width can regulate mass transfer in multiple ways [46]. By narrowing the channel near the test line (Figure 5A), the design increases local hydraulic resistance, slows fluid transport, and forces upstream analytes into a smaller reaction area. This produces both a longer residence time and a higher effective analyte concentration in the capture region. When combined with highly doped upconversion nanoparticles for SARS‐CoV‐2 nucleocapsid protein detection, this geometric LFA achieved an LOD of approximately 10 pg mL^−^^1^ and more than 100‐fold improvement over conventional colloidal‐gold‐ and europium nanoparticle‐based LFAs (Figure 5B). This example shows that macroscopic strip geometry can improve sensitivity by coupling local analyte enrichment with intensified signal generation before final readout.

**FIGURE 5:**
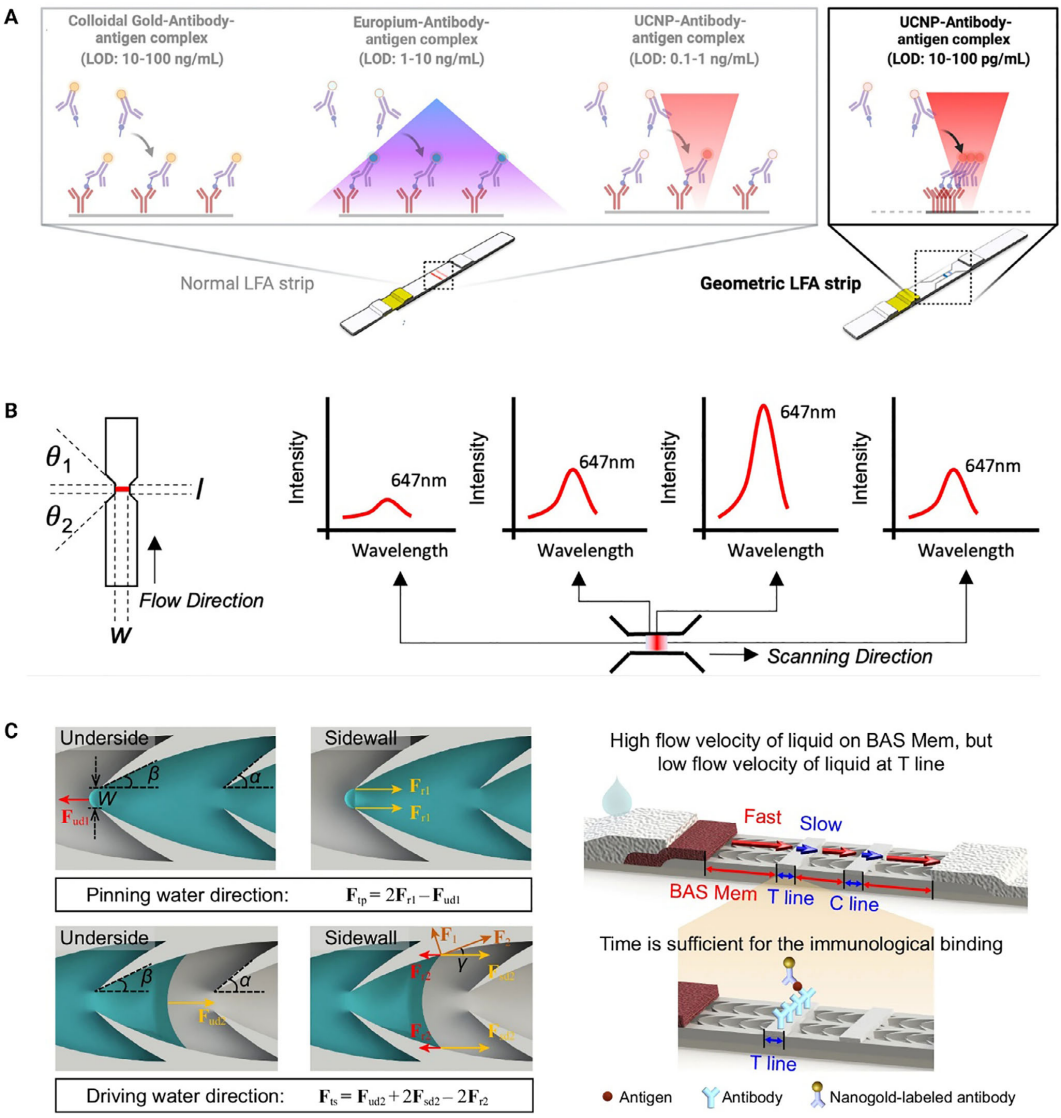
Regulating strip geometry and microfluidic flow fields for mass‐transfer optimization. (A) Comparison of analytical performance between conventional LFA architectures and a geometric “narrow‐gate” design. (B) Enhanced analytical performance achieved by combining narrow‐gate geometry with highly doped upconversion nanoparticles (UCNPs). Local flow constriction increases analyte concentration and residence time in the capture region, thereby lowering the limit of detection. Panels (A) and (B) are adapted with permission from Ref. [46]. Copyright 2024, ACS. (C) Surface‐guided rapid transport using a barbed arrow‐like structure membrane (BAS Mem). Interconnected asymmetric grooves and curved sidewalls generate directional capillary forces that promote rapid forward flow, suppress backward spreading, reduce residual liquid retention, and enable ultrafast cTnI detection. Adapted with permission from Ref. [77]. Copyright 2024, Springer Nature.

A related strategy is to extend the reaction window without permanently changing the membrane material. In the Lab‐on‐a‐Disc platform developed by Peshin et al. [76], LFA strips were integrated radially onto a rotating disc, and centrifugal force was used to counterbalance the capillary motion that normally drives chromatography. By adjusting rotational speed, the residence time of liquid in the detection zone could be extended from tens of seconds to several minutes. The platform also allowed a larger effective sample volume to pass through the strip. Compared with passive geometric narrowing, this approach provides dynamic control over the force balance governing transport. It is therefore particularly relevant for assays in which weak binding kinetics, low analyte abundance, or limited label brightness require more time for productive antigen–antibody capture.

However, not all diagnostic scenarios benefit from slower flow. For emergency biomarkers such as cardiac troponin I, shortening assay time while preserving sensitivity can be more important than simply increasing residence time. The barbed arrow‐like structure membrane (BAS Mem) reported by Li and co‐workers provides a different geometric solution to this problem [77]. Unlike conventional fiber‐based NC membranes, in which tortuous pores can trap liquid and retain detectable complexes outside the test and control lines, the BAS Mem uses interconnected asymmetric grooves bounded by curved sidewalls to impose directional capillary forces on the moving liquid (Figure 5C). These sidewall forces promote forward spreading while restricting backward or off‐path flow, giving the membrane a high flow‐rectification coefficient and much faster liquid transport than conventional NC membranes. When incorporated into cTnI lateral‐flow strips, the BAS Mem enabled detection within 240 s with a reported LOD of 1.97 pg mL^−^^1^, while reducing residual liquid retention, lowering background noise, and improving the signal‐to‐noise ratio. This case expands the concept of strip geometry from “slowing flow to improve binding” to “directing flow to improve speed, sample utilization, and background suppression.”

Together, these examples indicate that flow‐channel design should be matched to the dominant analytical bottleneck. Narrow‐gate structures and centrifugal platforms are most useful when assay performance is limited by insufficient local analyte concentration or inadequate reaction time, whereas surface‐guided rectifying structures are more suitable when the primary need is rapid transport, low residue, and fast readout. Thus, modifying strip geometry provides a complementary route to Phase I optimization: it can either intensify analyte–capture‐probe interactions or accelerate delivery through the strip, depending on the clinical and analytical demand. Future development of such strategies will need to balance sensitivity, speed, fabrication tolerance, sample viscosity, compatibility with large signal labels, and robustness in complex biological matrices.

## 4 Phase II: Reaction

After analytes and labeled probes are transported through the porous membrane during Phase I, they arrive at the detection zone, where the dominant constraints on assay performance shift from bulk transport to interfacial biochemical recognition. At this stage, labeled targets encounter capture receptors immobilized at the test line, and the final signal begins to depend less on how fast species move through the strip and more on how effectively they bind once they reach the interface. Although mass transfer remains relevant because it determines analyte supply and residence time, the decisive factors in Phase II are typically the accessibility of the reactive sites, the probability of productive recognition, and the stability of the complexes that form under flow. In practical terms, this means that efficient delivery alone is not sufficient: if the recognition interface is poorly organized or the resulting complexes are weakly stable, a substantial fraction of the transported target may still be lost to wash‐through, limiting both sensitivity and quantitative performance.

For this reason, the reaction phase is a key determinant of overall LFA performance. It is the point at which molecular recognition must be translated into retained surface‐bound complexes that can later generate measurable signal. In many assays, especially those targeting low‐abundance biomarkers such as circulating miRNAs and exosomes [21, 22, 23, 24], inflammatory or cardiac proteins such as IL‐6, procalcitonin, and cardiac troponin I [53, 78, 79], and urinary chemokines such as CXCL9/CXCL10 [80], the ability of the interface to capture and retain the analyte can become one of the main determinants of the practical limit of detection. This is also the stage in which compromises between kinetic efficiency, surface loading, and reaction time become most critical. Even when transport conditions are well controlled, assay performance may still be constrained by insufficient site accessibility, slow association under nonequilibrium flow, or dissociation of weakly retained complexes before signal readout. A useful way to frame these issues is through an interfacial kinetic description of complex formation.

In LFAs, target capture occurs under capillary flow within a finite residence time. Although the thermodynamic affinity of the recognition pair determines the stability of the complex, the signal generated during strip operation depends strongly on the rates of complex formation and dissociation before the sample front leaves the capture zone. If the surface interaction is approximated as a heterogeneous pseudo‐first‐order or second‐order binding process [81], the instantaneous rate of complex formation may be written as:

(5) $$\frac{dC_{AB}}{dt}=k_{assoc,eff}\times C_{A}\times \left(C_{B0}-C_{AB}\left(t\right)\right)-k_{disassoc}\times C_{AB}\left(t\right)$$

where *k*_assoc, eff_ is the effective forward association rate constant, *k*_disassoc_ is the dissociation rate constant, *C*_*B*0_ is the initial effective density of accessible binding sites at the test line, *C_AB_* (*t*) is the concentration of complexes already formed at time *t*, and *C_A_* (*t*) is the instantaneous concentration of analyte in the mobile phase. Although simplified, this expression is useful because it highlights the main variables available for reaction‐stage optimization. In general, sensitivity can be improved by increasing the accessible receptor density, enhancing the effective association rate through interface or probe engineering, reducing dissociation of bound complexes, or increasing the time available for the reaction to proceed, a factor already discussed in Phase I. Viewed in this way, the reaction stage provides a useful bridge between transport control and final signal generation, because it determines how much of the analyte delivered to the interface is ultimately retained and converted into detectable output.

The reaction stage is therefore shaped not only by the intrinsic affinity of a given recognition pair, but also by the way in which the interface is constructed and by whether the reaction scheme itself remains limited by the conventional stoichiometry of passive antigen–antibody binding. Recent work has addressed these constraints in several complementary ways. One direction has focused on improving how capture antibodies are presented on the membrane so that a larger fraction of their binding capacity remains accessible under flow conditions [81]. Another has sought to reconstruct the reaction pathway itself through enzymatic or catalytic amplification, thereby extending the effective sensitivity range beyond what can be achieved by passive binding alone. In addition, because reaction kinetics can also distort assay performance at the high‐concentration end, strategies to mitigate the hook effect have become increasingly relevant for maintaining monotonicity and broadening usable dynamic range.

### 4.1 Oriented Antibody Immobilization

For protein‐target LFAs, specific antigen–antibody recognition remains the core reaction mechanism. A typical immunoglobulin G (IgG) molecule adopts a Y‐shaped structure, with the Fab domains responsible for antigen binding and the Fc region providing structural support and mediating downstream interactions (Figure 6A) [82]. In most conventional LFA fabrication processes, capture antibodies are immobilized onto nitrocellulose or related substrates through passive adsorption or nonspecific chemical coupling. While operationally simple, this approach often produces random antibody orientation. Under such conditions, some Fab regions may face the membrane surface, become partially buried, or be sterically screened by neighboring molecules (Figure 6B). In addition, hydrophobic or electrostatic interactions with the substrate may perturb antibody conformation and reduce biological activity [83]. The consequence is that the effective density of accessible binding sites at the test line is often substantially lower than the nominal amount of antibody deposited. In kinetic terms, this deficiency in effective *C*_*B*0_ lowers the practical capture capacity of the interface and constrains the effective Damköhler number (*Da*) of the system, thereby reducing signal generation even when transport to the test line is adequate.

**FIGURE 6:**
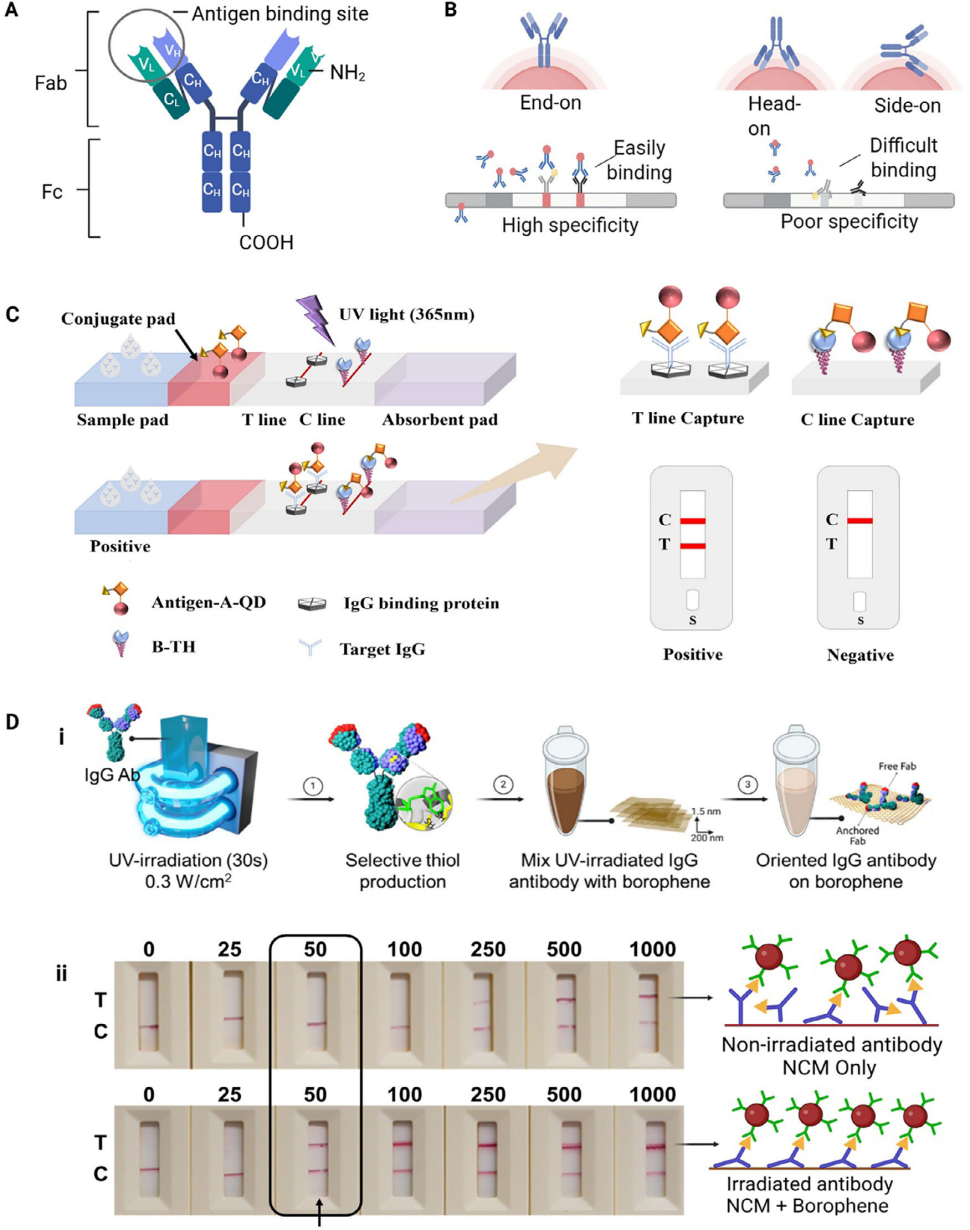
Strategies and functional effects of oriented antibody immobilization in Phase II. (A) Schematic illustration of the structure of a typical IgG antibody. (B) Influence of antibody orientation on interfacial capture efficiency and assay specificity. (C) Comparison between conventional LFA and affinity‐based LFA (AF‐LFA) for IgG detection. In AF‐LFA, high‐affinity binding pairs, such as SpyTag/SpyCatcher or StrepTag/Streptavidin, are used to promote oriented antibody immobilization and improve the accessibility of antigen‐binding domains. Reproduced with permission from Ref. [84]. Copyright 2025, Elsevier. (D) Oriented antibody immobilization via the photochemical immobilization technique (PIT). (i) Schematic illustration of borophene biofunctionalization, in which controlled UV irradiation selectively cleaves disulfide bonds to expose thiol groups, enabling directional covalent anchoring while preserving Fab‐region accessibility. (ii) Analytical performance comparison showing improved visual sensitivity for the oriented borophene–antibody conjugate LFA relative to a conventional format. Reproduced with permission from Ref. [85]. Copyright 2025, ACS.

Against this background, oriented antibody immobilization has emerged as an important strategy for improving reaction‐stage performance. The basic idea is to guide antibodies into a more uniform end‐on configuration, so that their Fab regions are preferentially exposed to the mobile phase while the Fc regions serve as anchoring points to the surface. In principle, this improves the efficiency of the reaction stage in two related ways: it increases the effective collision probability (increasing *k_assoc_*) between analytes and accessible binding domains, and it also increases the fraction of the deposited antibody population that remains functionally available for capture. Because these gains arise from interface organization rather than from increasing total protein loading, oriented immobilization is particularly attractive for LFA formats where the reaction zone is space‐limited and where efficient use of capture molecules is important.

The selection of recognition elements may also benefit from data‐driven screening before they are incorporated into the capture interface. For example, Yang et al. developed machine‐learning models that estimate antibody–protein antigen binding affinity from interface and surface descriptors [56]. Although this work was not conducted in an LFA format, it provides a relevant example of how AI/ML could help prioritize recognition pairs before experimental evaluation. In the context of LFAs, such screening may assist the selection of candidates expected to retain favorable binding behavior within the finite residence time imposed by capillary flow. Experimental characterization remains essential, particularly because affinity alone does not determine antibody orientation, surface accessibility, or performance in complex sample matrices.

A widely used route to such orientation is bioaffinity‐based immobilization. Protein A from *Staphylococcus aureus* and Protein G from *Streptococcus* bind specifically to the Fc region of mammalian IgGs and can therefore be used as molecular anchors on the strip surface. When these proteins are first immobilized on nitrocellulose or on the signal probe, subsequently introduced antibodies are more likely to adopt an Fc‐down, Fab‐out configuration. In one recent example, an engineered polymerized Protein G strategy was used to increase IgG‐binding capacity directly at the test line (Figure 6C). This arrangement enabled sensitive detection of SARS‐CoV‐2 nucleocapsid antibody in complex matrices such as saliva and blood, with a reported detection limit of 10 ng mL^−^^1^ [84]. Related affinity‐based approaches have also been built around the biotin–streptavidin interaction, whose dissociation constant is approximately 10^−^^1^ ^5^ M, representing one of the strongest non‐covalent interactions used in bioassay design. When antibodies are biotinylated at defined positions away from the Fab region and then captured on streptavidin‐coated surfaces, the resulting assemblies can provide improved stability and orientation. Because these orientation strategies modify the membrane‐bound recognition layer, their implementation should also preserve local wetting and allow flowing probe–target complexes to access the capture sites. Excessive surface coverage or poorly controlled interfacial modification could improve antibody orientation while creating an additional transport barrier near the test line.

Compared with these non‐covalent strategies, the photochemical immobilization technique (PIT) provides a covalent route to orientation control. Traditional crosslinking chemistries such as EDC/NHS generally rely on randomly distributed amino or carboxyl groups and therefore do little to control the final arrangement of the antibody on the substrate. PIT, by contrast, uses controlled ultraviolet irradiation to cleave disulfide bonds selectively in the hinge region of the antibody (Figure 6D, i). This exposes reactive thiol groups near the Fc region while largely preserving Fab integrity, allowing the antibody to anchor to suitably modified surfaces through a more directed and stable linkage. This strategy has recently been extended to the functionalization of two‐dimensional nanomaterials. In one example, photoinduced immobilization was used to couple capture antibodies directionally onto borophene nanosheets, producing an LFA platform for the endometriosis biomarker HMGB‐1 (Figure 6D, ii) [85]. In complex menstrual blood matrices, the assay reached a limit of detection of 40 pg mL^−^^1^ within 10 min, showing improved performance relative to conventional physically adsorbed strips. This study illustrates that improved orientation can translate directly into measurable gains in low‐abundance detection, especially when combined with a well‐defined nanostructured interface.

A more customizable route to orientation control is provided by recombinant fusion proteins. In this approach, antibodies, antibody fragments, or nanobodies are genetically fused to defined affinity motifs or reactive peptide tags, allowing their attachment to the substrate to be encoded at the molecular level. Among the best‐known systems is SpyTag/SpyCatcher, which forms an irreversible covalent isopeptide bond upon contact [86]. In LFA applications, capture antibodies can be expressed with a SpyTag sequence and then assembled onto beads or membrane surfaces bearing SpyCatcher. This provides a structurally well‐defined route to antibody presentation and reduces the risk of detachment during sample flow and wash‐through. In one study, single‐domain antibody dimers assembled through the SpyTag/SpyCatcher system improved dengue virus NS1 detection in 50% normal human serum over the tested concentration range of 1.4–89 ng mL^−^^1^, with the oriented capture format increasing NS1 detection by at least five‐fold compared with the non‐oriented format.

Analyzed from a physicochemical and kinetic perspective, oriented immobilization is not simply a surface‐functionalization technique, but a way of improving the functional organization of the solid–liquid reaction interface. By reducing steric shielding, increasing the accessible fraction of capture molecules, and stabilizing the geometry of the recognition layer, these methods can effectively increase *k*_on_, reduce apparent *k*_off_, and increase the practical density of functional binding sites. These changes are particularly important under low‐target conditions, where reaction efficiency rather than transport alone may determine whether a detectable signal is generated. By increasing the accessible receptor fraction and stabilizing the recognition geometry, oriented immobilization can shift the effective Damköhler balance toward a more reaction‐favorable regime and reduce the risk of false‐negative results under short‐residence‐time conditions. In this sense, oriented antibody immobilization provides a clear example of how Phase II engineering can improve LFA sensitivity without necessarily changing the signal label or the overall strip architecture. It also provides a useful conceptual foundation for the reaction‐amplification strategies discussed in the next section.

### 4.2 CRISPR‐Cas‐Mediated Reaction Amplification

In applications where LFAs are expected to detect extremely low‐abundance targets, the limitations of conventional passive capture formats become increasingly apparent. These targets include viral nucleic acids such as HPV16/18 DNA and SARS‐CoV‐2 RNA [87, 88], circulating molecular or protein biomarkers such as EV‐derived miRNAs, salivary miR‐31 and CXCL9/CXCL10 [80, 89, 90], and food‐safety contaminants or toxins such as procymidone, chloramphenicol and fumonisin B1 [91, 92, 93]. In such systems, signal formation can remain constrained by the stoichiometric nature of passive binding, so that sensitivity gains eventually approach a practical limit even when transport and capture interfaces are well optimized [94]. For this reason, considerable attention has turned to reaction‐stage strategies that do not simply increase the efficiency of binding, but reconstruct how target recognition is converted into a strip‐readable signal. Among these strategies, the integration of CRISPR‐Cas systems with LFAs has emerged as one of the most active directions. Rather than relying exclusively on one‐to‐one target capture at the test line, CRISPR‐LFA introduces target‐triggered enzymatic cleavage into the assay workflow, allowing a single recognition event to modulate many reporter molecules [95, 96].

The analytical value of CRISPR‐LFA arises from the collateral cleavage activity of certain Cas enzymes, which is distinct from their better‐known genome‐editing function. Diagnostic platforms such as SHERLOCK and DETECTR exploit the collateral nuclease activity of Cas13 and Cas12, respectively, and this mechanism was translated into a lateral‐flow‐compatible format through the SHERLOCKv2 architecture [96]. In many CRISPR‐LFA systems, the strip therefore reads out reporter cleavage rather than target capture alone. The upstream reaction may occur in a tube, an upstream pad, a separate incubation module, or an integrated paper‐based zone before the cleavage products are interpreted on the strip. The key advance is the insertion of catalytic turnover between molecular recognition and signal formation.

In most CRISPR‐LFA formats, this catalytic process is converted into a strip‐readable output using dual‐labeled reporter molecules, commonly bearing FAM and biotin at opposite ends (Figure 7A,i). In the absence of target, the intact reporter can bridge labeled anti‐FAM probes and streptavidin at the test line, producing a visible or instrument‐readable band. In the presence of target, collateral cleavage severs the reporter, disrupting this bridge and redistributing the probe signal, often producing a characteristic signal‐off response in which the test‐line signal decreases as target concentration increases. This format shifts interpretation from target accumulation alone to reporter‐cleavage kinetics.

**FIGURE 7:**
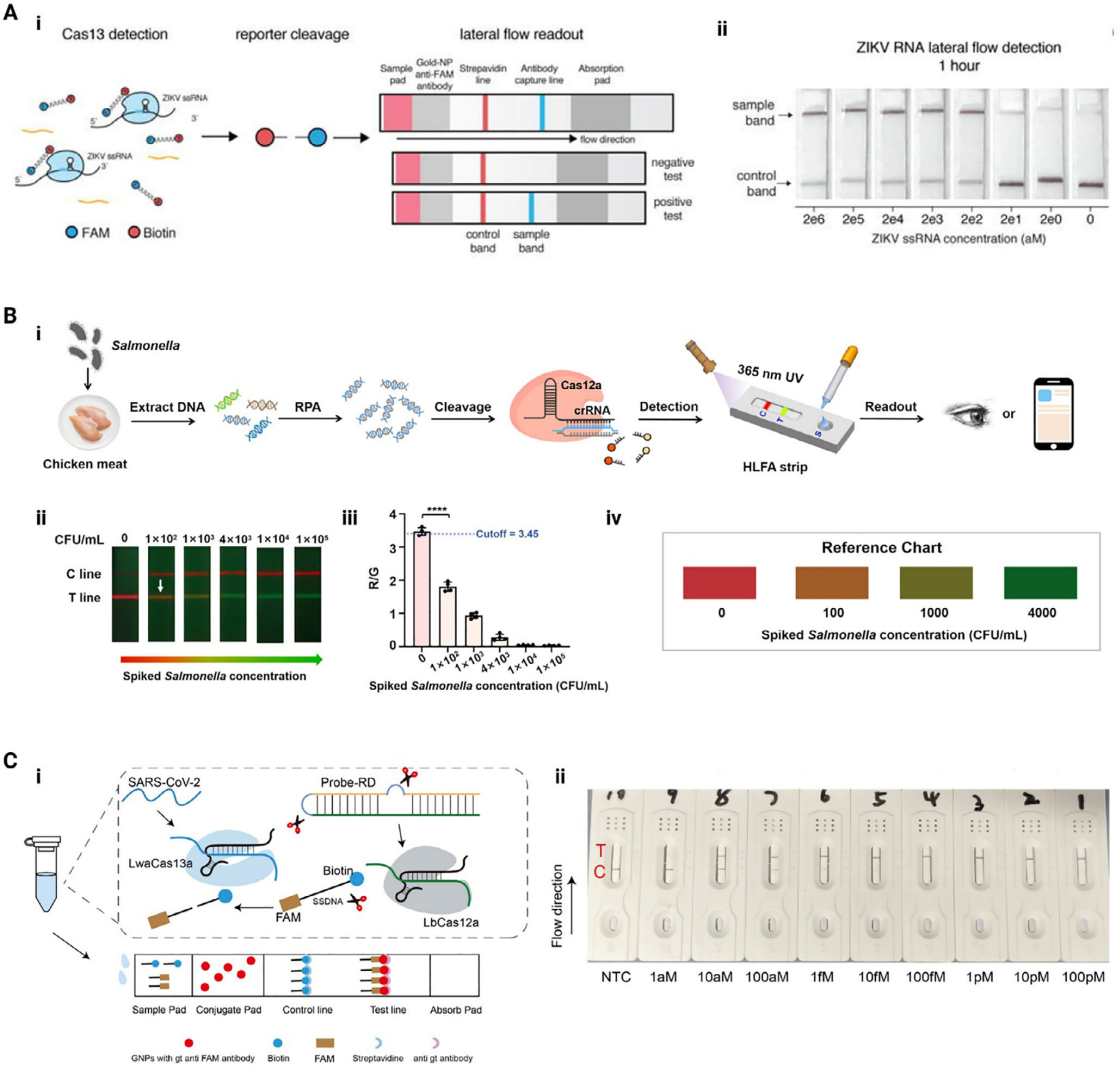
CRISPR‐Cas‐mediated reaction amplification and reaction‐network reconstruction in Phase II. (A) Fundamental architecture of a CRISPR‐mediated LFA, exemplified by SHERLOCK. Target recognition activates the collateral cleavage activity of the Cas enzyme, leading to cleavage of dual‐labeled reporter molecules and converting molecular recognition into a strip‐readable signal redistribution. Reproduced with permission from Ref. [96]. Copyright 2018, American Association for the Advancement of Science. (B) Optimization of readout kinetics using a hue‐recognition lateral flow assay (HLFA). Quantitative interpretation is based on dynamic fluorescence‐color evolution, expressed as the red/green (R/G) ratio, rather than absolute band intensity, thereby improving sensitivity to weak cleavage events and reducing masking effects caused by excess reporter substrate at low target concentrations. Reproduced with permission from Ref. [102]. Copyright 2024, ACS. (C) Amplification‐free cascade enzymatic reaction network. Tandem Cas13a–Cas12a cleavage shifts part of the amplification burden from upstream target pre‐amplification to the reaction network itself, enabling attomolar‐level detection without front‐end nucleic‐acid amplification. Reproduced with permission from Ref. [103]. Copyright 2024, ACS.

This strategy was initially developed primarily for nucleic‐acid analysis, but more recent studies have broadened its scope. Table 4 illustrates the range of formats that have emerged, including one‐pot EXTRA‐CRISPR detection of EV‐derived miRNAs with lateral‐flow compatibility and single‐digit femtomolar sensitivity [89]; orthogonal Cas12a/Cas13a multiplex LFA for HPV16 and HPV18, reaching 10 copies µL^−^^1^ [87]; and amplification‐free SERS‐assisted Cas12a detection of HIV‐1 dsDNA with single‐base specificity down to 0.3 fM [97]. Other examples include one‐pot Cas12a/ERA screening of 14 high‐risk HPV types at 50 copies reaction^−^^1^ [98] and a cascade nucleic‐acid amplification strategy for microRNA‐320d reaching 0.342 fM [90]. Importantly, this chemistry has also been extended to salivary miRNA detection. For example, IS‐primer amplification coupled with Cas12a enabled dual‐mode paper‐strip detection of salivary miR‐31, reaching 38.4 aM [90]. In another example, antibody–DNA barcoding was used to convert target proteins such as CXCL9 and CXCL10 into DNA readouts capable of triggering Cas12a cleavage, producing an LOD of 0.61 pg mL^−^^1^ [80]. Together, these examples show how CRISPR chemistry can link diverse recognition events to strip‐readable reporters.

**TABLE 4:** Representative CRISPR‐integrated LFAs and their reaction‐amplification strategies.

Target	CRISPR/reaction format	Readout strategy	Key design feature	Representative LOD	Practical consideration	Ref.
EV‐derived miRNAs, including miR‐21	One‐pot EXTRA‐CRISPR (RCA‐Cas12a)	Fluorescence and lateral‐flow readout	Padlock‐probe ligation, rolling‐circle amplification and Cas12a collateral cleavage	Single‐digit fM	Requires ligation/RCA/Cas12a coordination	[89]
HPV16, HPV18	Orthogonal Cas12a/Cas13a with RPA	Multiplex LFA	Dual‐enzyme multiplexing with reduced spatial crosstalk	10 copies µL^−^^1^	Requires upstream isothermal amplification	[87]
HIV‐1 dsDNA	Cas12a	SERS tag	Amplification‐free quantification with single‐base discrimination	0.3 fM	Requires Raman readout	[97]
High‐risk HPV types	Cas12a with ERA	Colorimetric LFA	One‐pot screening format	50 copies reaction^−^^1^	Workflow integration remains important	[98]
Salivary miR‐31	IS‐primer amplification coupled with Cas12a	Dual‐mode paper‐based strip	IS‐primer amplification with Cas12a‐mediated reporter cleavage	38.4 aM	Requires amplification design and strip optimization	[90]
Theophylline	dCas9 with allosteric transcription factor	LFA readout	Ligand‐responsive recognition beyond nucleic acids	2 µM	Lower sensitivity than nucleic‐acid formats	[99]
CXCL9, CXCL10	Cas12a with antibody–DNA barcode	Magnetic enrichment and LFA	Protein‐to‐DNA signal translation	0.61 pg mL^−^^1^	Requires antibody–DNA conjugation	[80]
miRNA‐10b	Cas12a with FCAS	Reverse fluorescence‐enhanced LFA	Reduces false positives in trans‐cleavage formats	5.53 fM	Requires optimized reporter design	[100]
Salmonella	Cas12a	Colorimetric/photothermal dual‐mode readout	Cross‐validating dual signal	1 CFU mL^−^^1^	Requires thermal readout for full sensitivity	[101]
SARS‐CoV‐2	dCas9 sequence recognition	Biotinylated LFA	Direct recognition without trans‐cleavage reporters	4 copies µL^−^^1^	Depends on sequence‐recognition efficiency	[88]

As the field has matured, it has become clear that performance in CRISPR‐LFA depends not only on the catalytic potential of the Cas enzyme, but also on how the reaction network and readout are tuned. One practical limitation is that excessive reporter concentration can mask weak cleavage events by creating a large substrate surplus. In response, readout strategies such as hue‐recognition lateral flow assays have been developed to analyze subtle fluorescence‐color changes rather than relying only on absolute band intensity (Figure 7B) [102]. This example illustrates that reaction‐stage amplification and signal‐stage interpretation are closely linked: the benefit of CRISPR cleavage is realized most effectively when the readout format is matched to the kinetics of the underlying enzymatic process. Another important direction is the reduction or elimination of front‐end nucleic‐acid pre‐amplification. Cascade Cas13a–Cas12a systems, for example, show that multiple enzymatic layers can be combined to shift part of the amplification burden from separate upstream PCR/RPA‐type steps into the reaction design of the assay itself (Figure 7C) [103]. Although such approaches still introduce operational complexity, they demonstrate how reaction‐stage engineering can partly compensate for the absence of conventional target amplification.

From a broader reaction‐stage perspective, CRISPR‐LFA extends the design logic of Section 4.1 by adding a catalytic layer between molecular recognition and strip readout. This catalytic layer increases signal generation per recognition event and is therefore particularly valuable for targets that are too scarce for passive binding alone. However, this gain also shifts part of the assay burden from simple strip chemistry to multicomponent enzymatic reaction networks. From a translational perspective, CRISPR‐LFAs are most attractive for high‐value infectious‐disease and molecular‐biomarker applications, but they remain less mature than conventional antigen strips. Their broader implementation is constrained by enzyme and guide‐RNA stability, contamination control, incubation temperature, sample preparation, and batch‐to‐batch activity of the reagent system. Advances in lyophilization, paper‐based reagent storage, and microfluidic automation may move these enzymatic cascades toward more self‐contained and field‐deployable formats. In the near term, however, CRISPR‐LFAs are more likely to translate into hospital POCT, specialist clinics, GP‐based molecular testing, or controlled field settings than into low‐cost home tests.

### 4.3 Mitigating Hook Effect

In sandwich‐format LFAs, the relationship between target concentration and signal intensity is not always monotonic. When the target concentration (*C_A_*) rises beyond a critical range, excess free target can rapidly occupy capture sites at the test line while simultaneously consuming a large fraction of the labeled detection antibodies in the flowing phase (Figure 8A). Under these conditions, the formation of the intended sandwich complex (*C_AB_*) becomes less efficient, and the measured signal may decrease despite a further increase in the true target concentration [104]. This phenomenon, commonly referred to as the high‐dose hook effect, reflects a stoichiometric imbalance within the reaction system [105, 106]. From the perspective of Phase II, the problem is not simply one of signal saturation, but of altered reaction ordering: when the concentration of free target becomes too high, the spatiotemporal sequence required for effective sandwich assembly is disrupted, and the relationship between target concentration and final readout becomes distorted.

**FIGURE 8:**
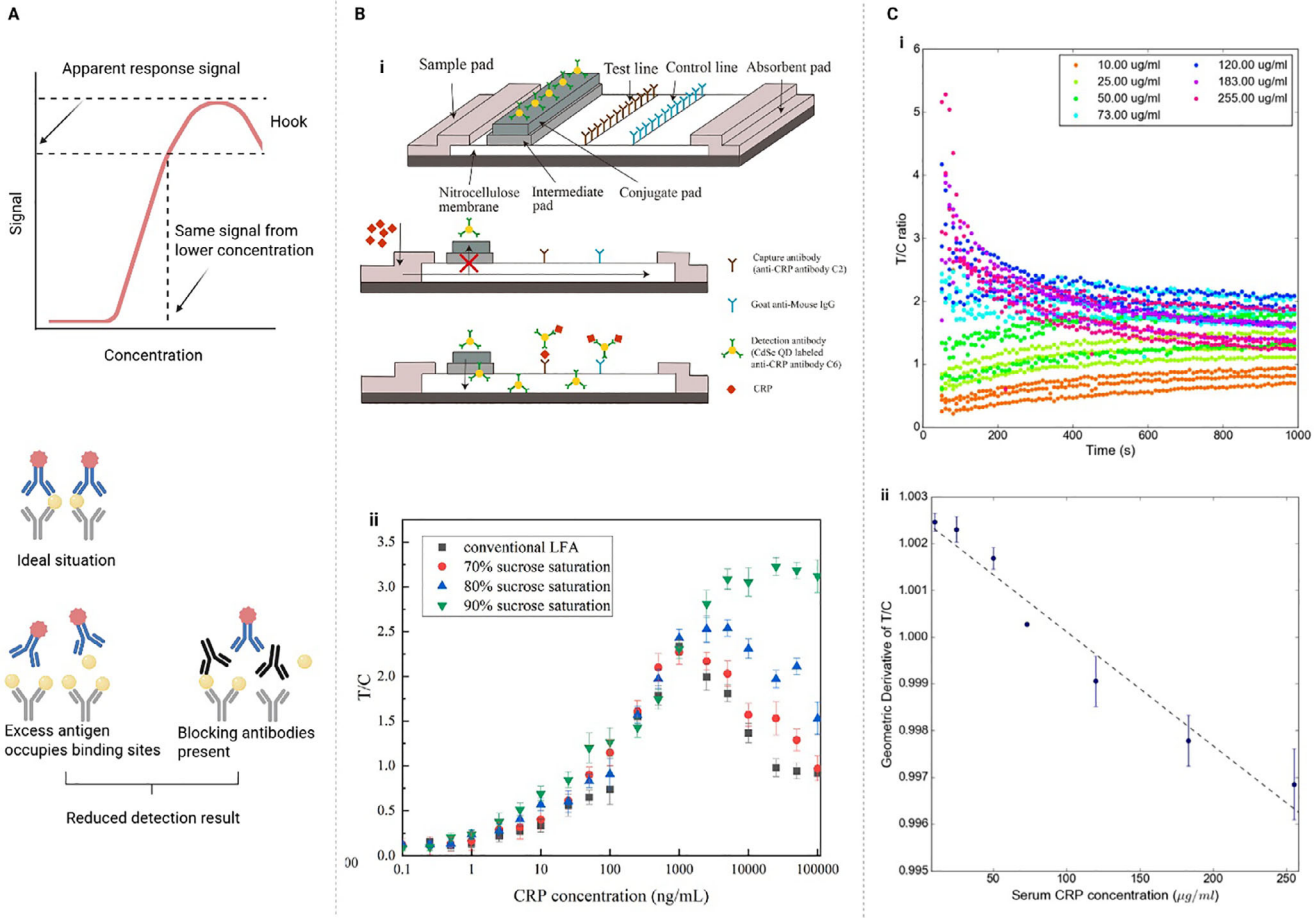
The high‐dose hook effect in LFAs and representative mitigation strategies in Phase II. (A) Schematic illustration of the high‐dose hook effect. At excessively high target concentrations, free antigens can saturate capture sites and competitively consume labeled detection antibodies, thereby disrupting sandwich‐complex formation and generating falsely low signals. (B) Mitigation through spatiotemporal regulation of transport. (i) Schematic of an LFA incorporating a sucrose‐treated intermediate pad. Upon dissolution, the sucrose layer forms a transient high‐viscosity barrier that delays the release of detection antibodies, thereby favoring sequential rather than competitive binding at the reaction interface. (ii) Corresponding calibration curves showing extension of the dynamic range and alleviation of hook‐effect behavior. Reproduced with permission from Ref. [107]. Copyright 2022, Elsevier. (C) Mitigation through early‐stage kinetic monitoring. (i) Real‐time evolution of the test/control (T/C) signal ratio at different concentrations of C‐reactive protein (CRP). (ii) Quantitative interpretation based on the early kinetic profile, enabling discrimination between genuinely low signals and false‐low signals caused by the hook effect before the assay reaches its endpoint. Reproduced with permission from Ref. [105]. Copyright 2017, ACS.

While sample dilution or increasing the concentration of detection antibodies are intuitive approaches, they can complicate the operational workflow of POCT. Manual dilution introduces the possibility of operator‐dependent error, while increasing probe concentration may elevate background and compromise sensitivity at the low‐concentration end. For this reason, more recent efforts have focused on strategies that preserve assay simplicity while restoring the reaction sequence needed for reliable sandwich formation. In practice, these strategies have generally followed one of two directions: physical regulation of transport order within the strip to reduce competitive binding, or early‐stage kinetic interpretation before the system reaches its misleading endpoint state. One physical approach is to introduce a controlled temporal delay between analyte arrival and probe arrival at the test line. For example, He et al. [107] achieved this by inserting a sucrose intermediate pad between the conjugate pad and the membrane (Figure 8B, i). As the sucrose dissolves, it forms a transient high‐viscosity barrier that slows the downstream movement of the labeled antibodies. This creates a sequential transport regime in which the unlabeled analyte reaches the test line first and binds to the immobilized capture antibodies, while the labeled detection antibodies arrive later. By partially separating these two binding events in time, the design reduces competitive saturation and delays the onset of the hook effect, thereby extending the dynamic range without requiring an additional dilution step (Figure 8B, ii).

A different approach is to avoid relying exclusively on the equilibrium or near‐equilibrium end point of the assay. In conventional LFA workflows, the strip is typically read after chromatography has been completed, which is often the stage at which hook‐induced signal inversion becomes apparent. Kinetic monitoring offers an alternative by analysing the early evolution of the signal before the system reaches its final saturated state. Erickson's team [105] developed a portable CMOS camera‐based platform to monitor the time‐dependent behavior of the strip during the initial reaction period. In this regime (t→0), the rate of signal development (dSignal/dt) remains approximately proportional to analyte concentration before the effects of saturation dominate. By tracking the temporal change in the T/C ratio (Figure 8C), the system could distinguish low‐concentration samples, which showed a rising T/C trajectory, from high‐concentration samples in the hook region, which displayed a declining trajectory as the system saturated rapidly. This approach allows “true low” signals to be distinguished from “false low” signals generated by the hook effect, thereby extending the usable concentration range without altering the physical structure of the strip.

The hook effect illustrates a limitation of premixed sandwich assays when they are applied over a wide concentration range. For clinically important targets such as CRP or hCG, where both very low and very high concentrations may need to be measured reliably, the reaction stage may benefit from designs that partially decouple the sequence of binding events, whether through soluble barriers, microfluidic delays, or other forms of spatiotemporal control. At the same time, the success of kinetic monitoring suggests that some limitations of the sandwich format need not be solved exclusively through chemistry or strip architecture. With increasing access to portable imaging and computational analysis, algorithm‐based correction provides an additional route for preserving dynamic range through software‐assisted interpretation rather than more complex consumable design. For clinical POCT, hook‐effect mitigation is therefore not merely a sensitivity issue, but an important reliability consideration for assays expected to operate across broad and unpredictable concentration ranges. In that sense, hook‐effect mitigation forms a useful transition between the reaction‐stage considerations discussed here and the signal‐processing strategies [105] discussed in the following section.

## 5 Phase III: Signal Transduction and Interpretation

Once specific binding has been established at the interface during Phase II, the LFA workflow enters the signal transduction stage. The role of this phase is to convert the microscopic population of molecular complexes (C_AB_) accumulated at the test line (T‐line) into a macroscopic signal (S) that can be extracted visually or instrumentally. In practice, however, the final analytical output is rarely determined by the number of complexes bound alone. It is also shaped by how efficiently the chosen probe converts molecular recognition into a measurable physical response, how strongly components contribute to background interference, and how much systematic or stochastic noise is introduced during signal acquisition. From this perspective, Phase III is not simply the endpoint of the assay, but the stage at which the analytical information generated upstream is either preserved, amplified, or partially obscured by the physics of the readout itself.

This conversion relationship can be parameterized through a phenomenological equation:

(6) $$S=\alpha \cdot C_{AB}+\beta_{bg}+N_{noise}$$

where *S* is the extracted signal, α is the signal transduction coefficient defined by the intrinsic physical properties of the probe (e.g., molar extinction coefficient, luminescence quantum yield, or Raman scattering cross‐section), *C_AB_* is the surface density of the complexes accumulated at the test line, β_*bg*_ represents optical, thermal, or electrochemical background contributions from the membrane and sample matrix, and N_noise_ denotes the systematic and stochastic fluctuations introduced during signal extraction. Although simplified, this expression helps clarify why improvements made in the earlier phases do not always translate directly into better assay performance. Stronger binding or more efficient analyte delivery can still yield only modest analytical gains if the probe itself produces weak signal, if the matrix generates substantial background, or if the readout system introduces high variability. The engineering challenge in Phase III is therefore not only to increase α, but also to suppress β_*bg*_ and reduce *N_noise_* while retaining the simplicity and portability that make LFAs useful in the first place.

Early strips relied mainly on the visible accumulation of probes on the membrane, and their analytical performance was therefore constrained by the threshold of human visual perception. The introduction of photoluminescent probes, including fluorescent microspheres and quantum dots, shifted signal extraction toward externally excited and spectrally separated readout, making quantitative analysis more feasible and reducing part of the matrix‐derived background [10]. Subsequent integration of higher‐order photophysical mechanisms, such as surface‐enhanced Raman scattering (SERS) and upconversion luminescence, further improved sensitivity by increasing signal specificity [108, 109]. These approaches utilize specific photophysical conversion efficiencies (α) to maximize signal intensity and further physically separate the characteristic signal from autofluorescence or other unwanted backgrounds (β_*bg*_). More recently, the growing use of smartphones, wearable platforms, and AI‐assisted analysis has extended Phase III beyond materials engineering alone, so that signal transduction is now increasingly shaped by the combined effects of probe design, reader architecture, and computational processing [33]. Algorithms and AI models are increasingly used to process physical signals directly, playing a critical role in compensating for ambient light interference, smoothing stochastic fluctuations, constructing nonlinear quantitative relationships, and recognizing complex signal patterns. As illustrated in Figure 9, the signal stage has gradually expanded from a simple visual endpoint into a readout stage involving optical, thermal, electrical, and digital components.

**FIGURE 9:**
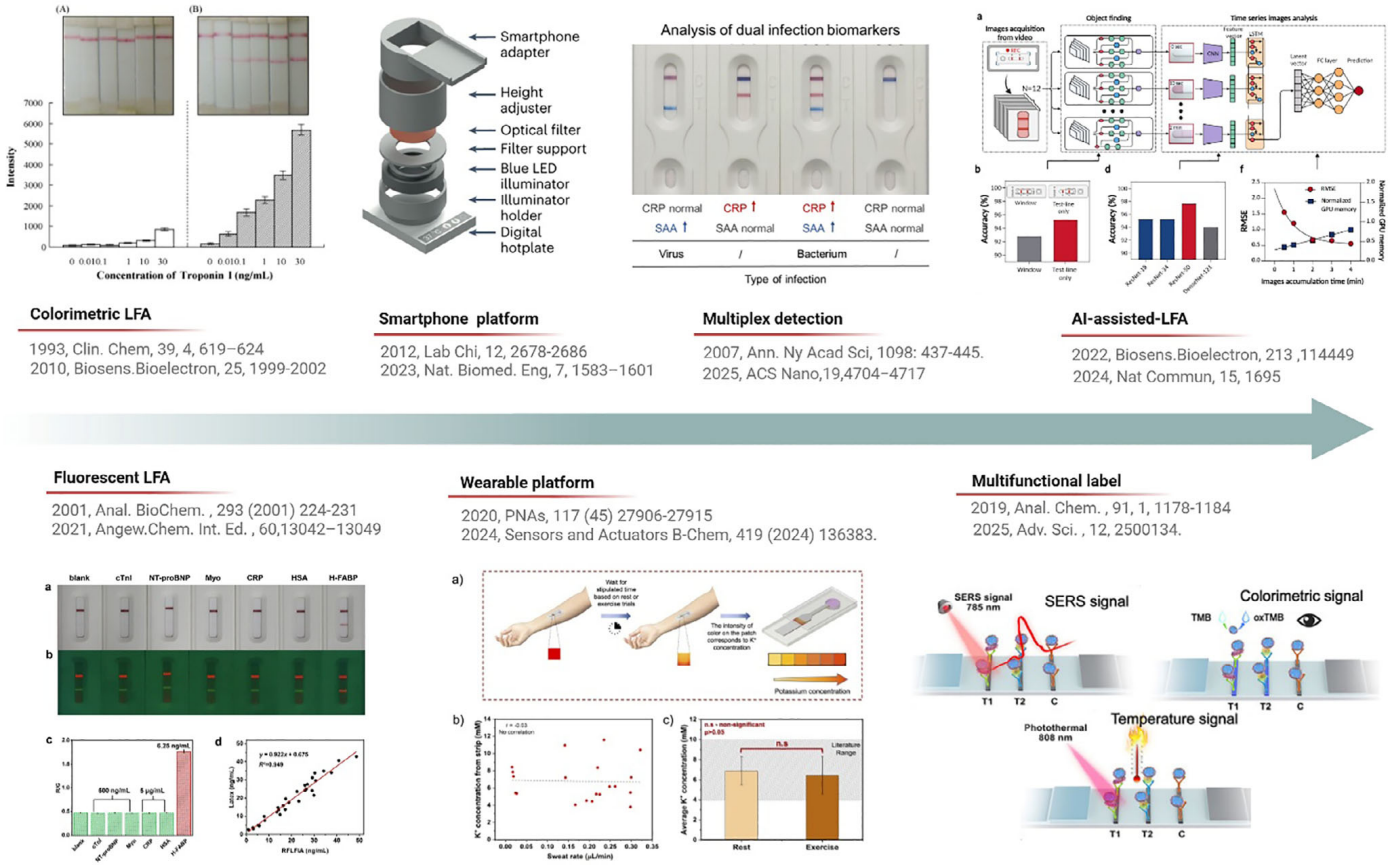
Evolution of signal transduction and interpretation in LFAs. The development of LFA signal transduction has progressively expanded from simple visual readout toward multi‐physical and digitally assisted interpretation. Early colorimetric and fluorescent formats established the basis for qualitative and semiquantitative detection through probe accumulation, optical absorption, and photoluminescence. With increasing demand for higher sensitivity, multiplexing, and matrix tolerance, signal strategies have expanded to include spectrally narrow probes, low‐background luminescent labels, and nonoptical modalities. More recently, smartphones, wearable platforms, and AI‐assisted analysis have further shifted Phase III from label‐centred signal generation toward integrated signal acquisition, noise correction, quantitative interpretation, and digital connectivity. Adapted from Refs. [89, 110, 111, 112, 113, 114, 115]. Refs. [89, 110, 111, 113, 114] are adapted with permission. Copyright 2023, Springer Nature; 2010 and 2024, Elsevier; 2025, ACS; and 2021, Wiley. Refs. [112, 115] are adapted under the terms of the Creative Commons Attribution 4.0 License. Copyright 2024 and 2025, The Author(s).

At the materials level, probe engineering has moved from conventional colorimetric labels to luminescent, narrow‐band, catalytic, and multimodal probes, aiming either to increase intrinsic transduction efficiency or to shift the signal into a less interference‐prone domain. At the hardware level, dedicated readers, smartphone peripherals, and wearable interfaces stabilize illumination, collection geometry, and thermal or electrical interrogation conditions, reducing variability during readout. At the computational level, machine learning and deep neural networks provide software compensation for spatial heterogeneity, weak signals, and nonlinear calibration behavior. In parallel, the need for multidimensional diagnostics has promoted spatial and spectral multiplexing, giving rise to multiplexed analytical architectures. The following sections discuss these developments in sequence, beginning with signal‐label engineering, which defines the core signal intensity.

### 5.1 Signal‐Generation Strategies: Labels and Transduction Mechanisms

Within the framework above, signal labels are the primary determinants of the intrinsic transduction coefficient α. They form the functional bridge between microscopic recognition at the interface and the macroscopic signal ultimately extracted by the user or instrument. By introducing defined physical response mechanisms, such as light absorption, photoluminescence, vibrational scattering, electron transfer, or photothermal conversion, nanoprobes translate molecular binding events into measurable features. Their importance lies not only in the magnitude of the signal they can generate, but also in the physical dimension through which that signal is expressed. In many cases, improvements in performance arise not simply because a probe is “brighter,” but because it shifts the readout into a regime that is less affected by the matrix background represented by β_*bg*_. For this reason, signal‐label engineering remains one of the most direct and influential routes for improving Phase III performance.

Table 5 summarizes how probes based on different transduction mechanisms differ in sensitivity, interference resistance, instrumentation requirements, and practical suitability. Conventional colorimetric labels such as AuNPs rely on visible absorption and scattering, remain attractive because they require no external excitation, and are well suited to routine screening and low‐cost decentralized testing, although their typical detection range is often around 1–10 ng mL^−^^1^. Nanozyme‐based catalytic colorimetric systems add substrate‐driven amplification and can often reach 1–100 pg mL^−^^1^ while retaining simple visual or smartphone‐readable formats. Fluorescent labels, including quantum dots, fluorescent microspheres, europium chelates, and FITC, typically reach roughly 10 fg mL^−^^1^ to 10 pg mL^−^^1^, but require more controlled readers. Upconversion luminescence and SERS tags can reach the 1 fg mL^−^^1^ to 1 pg mL^−^^1^ range, while electrochemical and photothermal labels offer low analytical limits when optical readout is hindered by opacity or scattering. Thus, signal‐label selection is not simply a materials choice: it determines reader requirements, user training, and realistic deployment settings, from home testing and remote clinics to GP surgeries and hospital‐based POCT. Across modalities, sensitivity gains often shift the limiting factor from probe accumulation to background suppression, calibration, reader control, or manufacturing reproducibility.

**TABLE 5:** Comparison of representative signal‐transduction modalities in LFAs.

Signal modality	Representative probes	Representative reported LOD range	Required reader	Main strengths	Main limitations	Feasible use settings
Aggregation‐based colorimetric	AuNPs	1–10 ng mL^−^^1^	Naked eye, smartphone, strip reader	Lowest cost; simplest workflow; rapid visual readout	Limited sensitivity; subjective interpretation near visual threshold	Home use; remote clinics; mass screening; pregnancy and infectious disease antigen tests
Catalytic colorimetric	Nanozymes, Pt, Fe_3_O_4_, Prussian blue, Co–N–C with TMB/H_2_O_2_	1–100 pg mL^−^^1^	Naked eye, smartphone, simple optical reader	Signal amplification while retaining visual readout	Additional substrate step; possible matrix interference	GP clinics; remote clinics; assisted home testing; higher‐sensitivity visual POCT
Fluorescence	QDs, fluorescent microspheres, Eu chelates, FITC	10 fg mL^−^^1^–10 pg mL^−^^1^	Portable fluorescence reader or UV/LED excitation module	High sensitivity; quantitative readout	Autofluorescence; excitation and filtering required	GP clinics; pharmacies; community clinics; hospitals; reader‐assisted quantitative POCT
UCNPs	NaYF_4_:Yb/Er, NaYF_4_:Yb/Tm	1 fg mL^−^^1^–1 pg mL^−^^1^	NIR laser with portable optical reader	Low autofluorescence; good matrix tolerance; multiplexing potential	Requires NIR excitation; thermal management and reader standardization	Hospitals; specialist clinics; high‐value GP POCT; complex‐matrix or low‐abundance biomarkers
SERS	Au/Ag core–reporter–shell tags, nanostars, gap‐enhanced probes	1 fg mL^−^^1^–1 pg mL^−^^1^	Portable Raman spectrometer	Narrow spectral fingerprints; high multiplexing capacity; sensitive spectroscopic readout	Hotspot heterogeneity; Raman reader required	Hospitals; specialist diagnostics; reference‐lab‐adjacent POCT; multiplexed high‐sensitivity assays
Electrochemical	HRP/GOx enzymes, redox probes, screen‐printed electrodes, conductive nanomaterials	1 fg mL^−^^1^–100 pg mL^−^^1^	Portable potentiostat or integrated electrode reader	Resistant to optical color/scattering interference; compatible with electronics	Electrode integration and contact stability	GP clinics; hospitals; remote clinics with portable readers; colored or turbid samples
Photothermal	AuNRs, gold nanostars, Pd–Au nanoplates, carbon nanotubes, IR dyes	10 fg mL^−^^1^–1 pg mL^−^^1^	NIR laser and infrared thermal imager or temperature sensor	Strong matrix tolerance; dual visual/thermal readout possible	Requires thermal reader; environmental temperature control	Hospitals; specialist POCT; controlled field settings; scattering or pigmented matrices

*Note*: The LOD ranges are representative values reported in selected high‐sensitivity studies and should be interpreted as approximate performance windows rather than universal limits for each modality.

#### 5.1.1 Optical Signal Labels

Optical labels remain the most established class of signal probes in LFAs because they provide a direct physical link between interfacial molecular recognition and macroscopic signal readout. Through photon absorption, scattering, or radiative emission, these probes convert binding events at the test line into visible or instrument‐detectable optical features. Optical probes also have relatively low hardware dependence: colorimetric probes based on broad‐spectrum absorption enable passive qualitative assessment via visual contrast, while fluorescent probes relying on spectral separation facilitate feature extraction and quantification through portable optoelectronic components (e.g., CMOS sensors). Their continued importance reflects not only the maturity of optical detection technologies, but also the wide range of physical mechanisms available within this category. At the simplest end, broad‐band absorption enables equipment‐free visual interpretation, whereas more advanced luminescent and spectroscopic probes allow signal extraction to be shifted into less interference‐prone optical domains. Optical‐label engineering acts mainly by increasing the intrinsic transduction coefficient α and, in many cases, by reducing the effective contribution of matrix‐derived background β_*bg*_. On this basis, Figure 10 outlines the primary physical architectures of optical labels in LFAs, which can be broadly considered in three groups: colorimetric probes based on absorption and scattering, photoluminescent probes based on fluorescence or upconversion emission, and SERS probes based on vibrational fingerprint extraction under localized electromagnetic enhancement.

**FIGURE 10:**
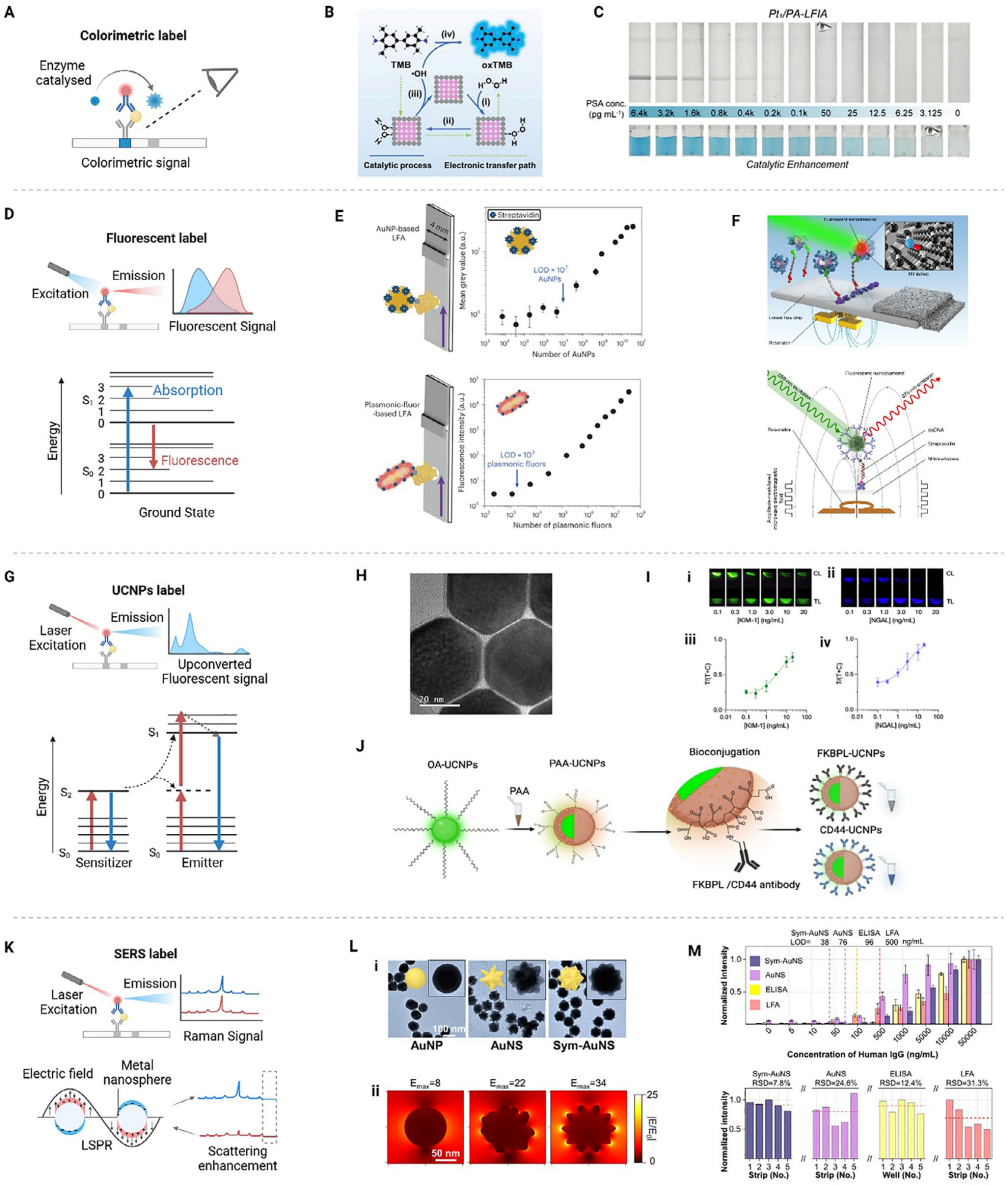
Optical labels and their signal‐generation mechanisms in LFAs. (A, D, G, K) Schematic illustrations of representative optical signal mechanisms in LFAs, including nanozyme‐assisted colorimetric amplification, fluorescence, upconversion luminescence, and surface‐enhanced Raman scattering (SERS), respectively. (B, C) Nanozyme colorimetric labels. (B) Signal‐generation mechanism based on nanozyme‐catalyzed TMB color development. Reproduced with permission from Ref. [52]. Copyright 2023, ACS. (C) Analytical performance of Pt_1_/PA‐based LFA for carcinoembryonic antigen (CEA) detection, showing visual test results before and after catalytic color development. Reproduced with permission from Ref. [119]. Copyright 2025, Elsevier. (E, F) Fluorescent labels. (E) Comparison of test‐site signals obtained using conventional streptavidin‐conjugated AuNPs and plasmonic fluors, with signal outputs represented by mean grey values and fluorescence intensities, respectively. Insets show schematic illustrations of the nanoconjugates. Reproduced with permission from Ref. [78]. Copyright 2023, Springer Nature. (F) Fluorescent nanodiamond (FND)‐based LFA. Microwave modulation of nitrogen‐vacancy centre fluorescence enables frequency‐domain separation of the probe signal from static background fluorescence. Reproduced with permission from Ref. [126]. Copyright 2020, Springer Nature. (G–J) UCNP labels. (G) Schematic illustration of upconversion luminescence signal generation in LFAs. (H) TEM image of UCNPs. Reproduced from Ref. [127], with permission from the ACS. (I) Photographs of membrane sensors and calibration curves for KIM‐1 and NGAL detection. Reproduced with permission from Ref. [128]. Copyright 2024, ACS. (J) Schematic illustration of UCNP modification for detection of FKBPL and CD44 in plasma samples from healthy and early‐onset preeclampsia patients. Reproduced with permission from Ref. [47]. Copyright 2023, Wiley‐VCH. (L, M) SERS labels. (L) TEM images (i) and corresponding electric field simulations (ii) for AuNPs, traditional Au nanostars (AuNS), and highly symmetric Au nanostars (Sym‐AuNS). (M) Comparison of the limit of detection (LOD) and relative standard deviation (RSD) for human IgG detection in human serum. Reproduced with permission from Ref. [49]. Copyright 2024, ACS.

##### 5.1.1.1 Colorimetric Labels

Colorimetric probes remain the most widely used optical labels in LFAs, largely because they combine operational simplicity with minimal hardware requirements. In conventional strips, AuNPs generate signal through localized surface plasmon resonance (LSPR)‐mediated absorption and scattering after accumulating at the test line [116]. This mechanism has been central to the commercial success of LFA technology, but it also introduces a practical sensitivity limitation. In practice, a probe aggregation density of approximately 10^8^ particles mm^−^^2^ at the test line is typically required before the band becomes readily visible to the naked eye. This absolute threshold helps explain why classical AuNP‐based LFAs often remain limited to the ng mL^−^^1^ range. One way to extend performance without simply increasing the number of captured particles has been to improve the optical response of each individual probe. Compared with isotropic spherical particles, anisotropic gold nanostructures such as nanorods, nanostars, and nanoclusters generate stronger localized electromagnetic coupling and higher molar extinction coefficients, thereby increasing the effective contribution of each particle to the overall colorimetric signal [117]. In this sense, topological control of plasmonic probes offers a direct route to increasing α without depending solely on greater probe accumulation. The principal advantage of conventional AuNP‐based colorimetric LFAs is therefore not maximal sensitivity, but their compatibility with equipment‐free interpretation, dry storage, low‐cost manufacturing and decentralized use. However, the optical advantage of larger or more structurally complex probes should be balanced against their behavior during chromatography. Changes in hydrodynamic size, surface chemistry, or conjugation density can alter probe migration through the membrane and access to the capture interface, thereby affecting the amount of label ultimately available for signal generation.

When analyte concentrations move further into the pg mL^−^^1^ regime, however, purely accumulation‐based colorimetry begins to approach the lower practical limit of optical absorption. Under these conditions, chemically catalytic labels, particularly nanozymes, provide an alternative signal‐generation mechanism (Figure 10A). Rather than serving only as passive optical absorbers, captured nanozymes catalyze the conversion of subsequently introduced substrates such as 3,3',5,5'‐tetramethylbenzidine (TMB) or 3,3'‐diaminobenzidine (DAB) into colored products, thereby transforming one interfacial binding event into a growing population of chromogenic molecules (Figure 10B) [52, 118]. This shifts the signal logic from physical accumulation to post‐capture chemical amplification and makes the colorimetric response less tightly tied to the number of retained particles alone. The design of such probes has also become increasingly refined. In a Pt_1_/PA single‐atom nanozyme system, for example, atomic‐level control over the local electronic environment of Pt was used to improve peroxidase‐like activity, leading to limits of detection of 1.21 pg mL^−^^1^ for carcinoembryonic antigen (CEA) and 0.6 pg mL^−^^1^ for prostate‐specific antigen (PSA) (Figure 10C) [119]. These examples suggest that colorimetric LFAs can partly overcome the sensitivity limits associated with simple optical accumulation, provided that the mechanism of signal generation itself is redesigned. The main trade‐off is that catalytic amplification improves colorimetric sensitivity but may introduce additional substrate handling, timing control, and dry‐state stability requirements that must be reconciled with the one‐step workflow expected of LFAs.

##### 5.1.1.2 Photoluminescence Labels

Photoluminescent probes provide a second major class of optical labels and are often favored when the assay needs to move beyond visual screening toward more reliable quantification. In these systems, the probe absorbs external excitation energy and emits photons at a defined wavelength, allowing the signal to be separated, at least in part, from the background through excitation‐emission discrimination [120, 121]. This provides a stronger foundation for quantitative readout than simple color contrast and has made fluorescent dyes [121], fluorescent microspheres [122, 123], and semiconductor quantum dots [124, 125] important components of reader‐assisted LFAs. At the same time, conventional fluorescence also faces a well‐known limitation. Because many fluorescent probes operate with relatively small Stokes shifts, short‐wavelength excitation can simultaneously stimulate autofluorescence from serum, whole blood, and even the nitrocellulose membrane itself. This raises the effective background term and can obscure weak signals associated with very low‐abundance targets (Figure 10D). In practice, the analytical value of conventional fluorescent probes therefore depends not only on how strongly they emit, but also on how effectively their signal can be distinguished from co‐excited background.

One route to improving fluorescence‐based LFAs is to increase the intrinsic radiative output of the probe so that the characteristic signal dominates the static background. Plasmonically enhanced fluorescence provides a representative example of this approach. Gupta et al. constructed a plasmonic fluor based on gold nanorods (AuNRs), in which near‐field enhancement and a Purcell‐effect‐driven increase in radiative decay accelerated the emission process of adjacent fluorophores [78]. In practical testing, this design lowered the limit of quantification for IL‐6 to 93 fg mL^−^^1^ (Figure 10E). While this signal‐dominant strategy can substantially improve analytical sensitivity, it does not remove the source of autofluorescence itself and may still be influenced by co‐excitation of unwanted species in the matrix. For this reason, a second line of development has focused more directly on physically decoupling the signal from the background.

A particularly instructive example of decoupling is provided by fluorescent nanodiamonds containing nitrogen‐vacancy (NV) centres. In these probes, fluorescence can be actively modulated through the spin‐state physics of the NV centre, allowing the target‐related signal to be extracted in the frequency domain. Miller et al. used microwave excitation together with lock‐in amplification to isolate the modulated fluorescence from the constant autofluorescence background (Figure 10F) [126]. Using this approach, HIV‐1 p24 protein was detected with a limit of detection of 2.88 fg mL^−^^1^. The significance of this strategy lies not only in the absolute sensitivity achieved, but also in the fact that the signal is made distinguishable through dynamic modulation rather than through brightness alone. In other words, the gain arises from a more favorable separation between αand β_bg_, rather than simply from a stronger optical emitter.

UCNPs can provide another useful approach to reducing fluorescence‐background interference, particularly in biological settings [127]. Such probes rely on anti‐Stokes luminescence under near‐infrared excitation and are typically based on a hexagonal β‐NaYF_4_ host lattice sensitized by ions such as Yb^3^^+^ and activated by Er^3^^+^ or Tm^3^^+^ (Figure 10G,H). Because most biological matrices and porous membrane materials show minimal absorption and secondary emission in the near‐infrared region, excitation around 980 nm provides a low‐background optical window. In practical terms, this substantially reduces autofluorescence and makes UCNPs particularly attractive for highly complex matrices. Ghorbanpour et al. showed that UCNP‐based assays could maintain pg mL^−^^1^‐level quantitative sensitivity in patient plasma (Figure 10J) [47]. In addition, spatial co‐doping of different activators can create orthogonal emission channels under a single excitation source. Arai and co‐workers used this principle to achieve dual‐channel quantification of kidney injury molecule‐1 (KIM‐1) and neutrophil gelatinase‐associated lipocalin (NGAL) in urine, with limits of detection of 0.28 and 0.23 ng mL^−^^1^, respectively (Figure 10I) [128]. UCNPs reduce matrix‐derived autofluorescence and support low‐background detection, but their broader use depends on compact NIR excitation, thermal management, and standardized optical calibration. Commercial activity from upconversion‐based diagnostic companies such as Hotgen and Uniogen suggests growing translational interest, although adoption will depend on clear use cases where low‐background detection justifies the added reader complexity.

##### 5.1.1.3 Surface‐Enhanced Raman Scattering Labels

Surface‐enhanced Raman scattering (SERS) probes constitute a third major optical route and differ fundamentally from both colorimetric and photoluminescent systems in how they encode information. Rather than relying on broad‐band absorption or emission, SERS extracts narrow molecular vibrational fingerprints, which makes it particularly attractive for multiplexed assays and for situations in which broad optical spectra would lead to severe overlap. In these probes, the effective signal transduction coefficient α depends strongly on the localized electromagnetic hotspots generated by plasmonic nanostructures, since these hotspots amplify the Raman response of nearby reporter molecules by orders of magnitude (Figure 10K). At the same time, the porous and chemically heterogeneous environment of an LFA strip presents a challenge for SERS‐based readout. Exposed metallic surfaces are vulnerable to nonspecific adsorption, competitive desorption of Raman reporters, and interfacial instability, all of which increase signal variability and therefore contribute to the N_noise_ term.

To improve stability, many SERS tags are designed as core–reporter–shell structures, such as SiO_2_@Ag or AgMBA@Au, in which the reporter is confined to a protected near‐field region and the external shell reduces interference from the surrounding biochemical matrix. Once interfacial stability has been improved, further gains can be obtained through structural control of the plasmonic core. Highly branched anisotropic particles are particularly effective in this regard because sharp tips create strong local field enhancement through the lightning‐rod effect. Wu et al. exploited this principle using highly symmetric gold nanostars (Sym‐AuNS), which generated a denser and more uniformly distributed hotspot network on the probe surface (Figure 10L) [49]. In serum‐spiked assays for human IgG, this design yielded a limit of detection of 38 ng mL^−^^1^, while improving sensitivity by 2‐, 3‐, and 13‐fold relative to conventional asymmetric gold nanostars, ELISA, and standard LFAs, respectively (Figure 10M). At the same time, the relative standard deviation was reduced from 24.6% to 7.75%, indicating an improvement in signal reproducibility. This example illustrates that optimization of SERS labels in LFAs depends not only on generating a stronger signal, but also on producing a more stable and spatially uniform enhancement environment across the strip. Despite their analytical advantages, SERS‐LFAs remain more challenging to standardize than colorimetric or fluorescence‐reader formats. SERS provides narrow spectral fingerprints and strong multiplexing capacity, but its practical use remains constrained by Raman reader requirements, nanotag reproducibility, hotspot heterogeneity, spectral calibration, and reader‐to‐reader transferability.

Taken together, these examples show that optical‐label engineering in LFAs has moved from simple contrast generation to more selective and physically decoupled signal transduction. In early colorimetric strips, performance depended mainly on whether probe accumulation at the test line exceeded the threshold of visual recognition. Modern probes instead alter the physics of signal generation through catalytic amplification, enhanced radiative decay, orthogonal excitation, frequency‐domain modulation, or narrow‐band vibrational readout. These mechanisms make analytical signals more distinguishable from background and allow Phase III to partially offset upstream limits in target abundance, transport, or capture efficiency. Optical probes therefore do more than passively report events from Phases I and II. They also determine whether captured events can be read reliably under realistic sample and device conditions. For high‐performance LFAs, especially in complex matrices, optical‐label design can improve sensitivity beyond simple increases in captured‐probe number. Near the practical detection limit, background suppression and signal stability often become decisive.

#### 5.1.2 Nonoptical Signal Labels

When LFAs are applied to strongly scattering or absorbing matrices, such as whole blood, tissue homogenates, or undiluted saliva, signal extraction based purely on photon transmission can become increasingly difficult. Under these conditions, further improvement does not necessarily come from making the optical probe brighter; it may instead require shifting the readout into a different physical domain. Nonoptical labels follow this logic by introducing signal mechanisms based on electron transfer or thermal dissipation, thereby reducing the dependence of the characteristic response on sample transparency. In the framework of Phase III, such strategies are particularly relevant when the main limitation lies in the background term β_*bg*_ associated with optical attenuation or spectral interference. Rather than attempting to suppress that background within the same optical channel, nonoptical labels provide an alternative by moving the readout into a different transduction mode.

##### 5.1.2.1 Electrochemical Labels

Electrochemical labels provide one of the clearest examples of this shift in detection. In electrochemical LFAs (eLFAs), the signal is derived not from light but from charge‐transfer processes occurring at or near integrated electrodes within the chromatographic network [129]. Depending on the specific design, these systems can monitor changes in interfacial resistance, transient current, or the consumption and production of electroactive species generated during biorecognition (Figure 11A). Because the readout no longer depends on optical transparency, eLFAs are well suited to matrices that are opaque, turbid, or highly colored. At the same time, this advantage introduces a different engineering challenge, as integrating rigid electrodes with a soft nitrocellulose membrane creates mechanical discontinuities within the strip, and these interfaces can disturb chromatographic flow or generate fluctuations in contact resistance. In Phase III terms, the problem is no longer one of optical background, but of electrical instability and increased N_noise_, which can limit quantitative reproducibility from strip to strip.

**FIGURE 11:**
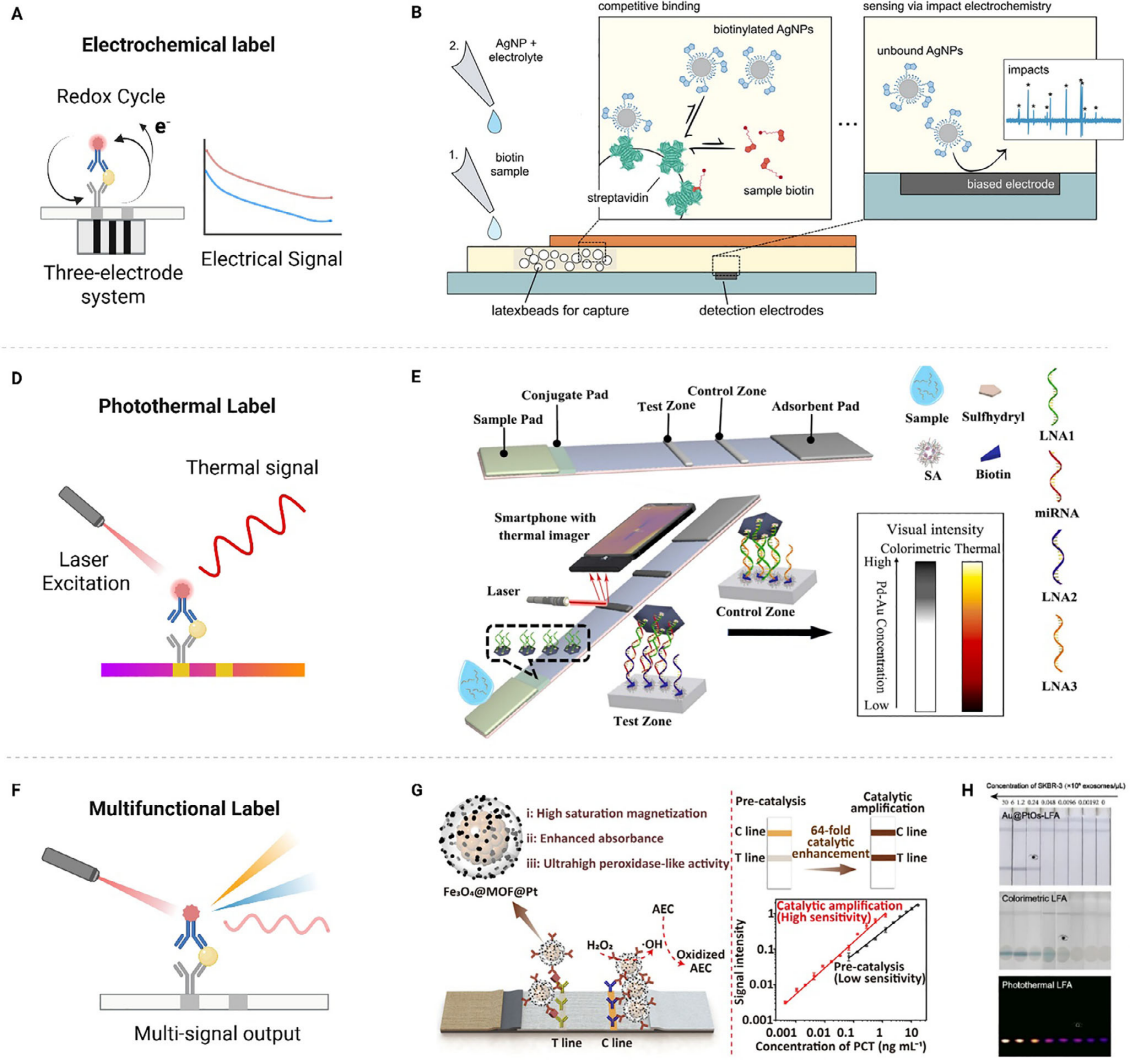
Nonoptical and multimodal labels for signal transduction in LFAs. (A–C) Electrochemical labels. (A) Schematic illustration of the basic principle of electrochemical LFAs. (B) Digital lateral flow sensor based on competitive binding and stochastic‐impact electrochemistry. Excess biotinylated nanoparticles are detected downstream through electrooxidative impact events at biased microelectrodes. (C) Calibration curve and temporal evolution of the mean impact rate, showing event‐based electrochemical quantification. Reproduced with permission from Ref. [131]. Copyright 2022, ACS. (D–E) Photothermal labels. (D) Schematic illustration of photothermal LFA readout. (E) Preparation and chain‐hybridization strategy of Pd–Au nanoplates for miRNA detection, showing the relationship between probe concentration and colorimetric/thermal outputs. Reproduced with permission from Ref. [133]. Copyright 2023, Elsevier. (F–H) Multimodal labels. (F) Schematic illustration of the basic mechanism of multimodal labels. (G) Schematic of the Fe_3_O_4_@MOF@Pt immunolabeled LFA. Reproduced with permission from Ref. [53]. Copyright 2022, ACS. (H) Au@PtOs driven LFA for multimodal detection of SKBR‐3 exosomes, showing SERS, catalytic colorimetric, and photothermal readouts at different exosome concentrations. Reproduced with permission from Ref. [134]. Copyright 2024, ACS.

Several recent studies have therefore focused less on electrochemical chemistry alone and more on how the electrical interface is constructed. A representative example is the in situ manufacturing strategy proposed by Calucho et al., who used CO_2_ laser direct writing to convert graphene oxide into highly conductive reduced graphene oxide (rGO) while simultaneously patterning the nitrocellulose membrane [130]. This monolithic, noncontact integration approach removes the conventional mechanically spliced boundary between electrode and membrane, while preserving the intrinsic capillary behavior of the strip. As a result, the system achieves more stable and lower‐impedance electrical contact, reducing physical fluctuations introduced during signal extraction. In this case, improved analytical performance is linked not only to the electrochemical principle itself, but also to more controlled integration of the electrode with the porous substrate.

A further development has been the redesign of the electrochemical readout logic itself. Wolfrum and co‐workers introduced stochastic impact electrochemistry into the lateral‐flow format, thereby shifting the readout from an ensemble signal to a series of discrete events [131]. In this architecture, excess biotinylated silver nanoparticles are detected downstream through the transient current pulses generated when individual particles strike a biased microelectrode (Figure 11B). Quantification is then based on counting these impacts rather than averaging a continuous signal, which reduces the influence of background Faradaic current. Using this digital electrochemical approach, free biotin could be quantified within 5 min over a dynamic range of 10 pM to 100 nM (Figure 11C). This “digital” quantification mechanism operating at the fundamental physical level can reduce interference from background Faradaic currents, baseline drift, and analogue noise.

From a systems perspective, electrochemical labels also offer an attractive route toward integration with flexible electronics. As electrochemical sensing elements are naturally compatible with microelectronic circuitry, eLFAs can more readily extend beyond the disposable strip format than many optical systems. A recent example is the development of wearable electrochemical LFA patches for epidermal sweat analysis [132]. In such devices, microfluidic channels, sweat–stimulation elements, and electrochemical sensing arrays are integrated on a flexible substrate, enabling real‐time in situ monitoring of inflammatory markers such as C‐reactive protein. This suggests that nonoptical transduction does not simply provide an alternative to optical readout in difficult matrices; it also aligns well with hardware formats that are relevant to continuous or wearable sensing.

##### 5.1.2.2 Photothermal Labels

Photothermal labels provide another nonoptical route for signal transduction, particularly in matrices where optical attenuation becomes difficult to avoid. Instead of extracting information from transmitted or emitted photons, these systems rely on non‐radiative decay processes that convert absorbed excitation energy, often in the near‐infrared, into localized heat (Figure 11D) [50]. Because the subsequent signal is read thermally rather than optically, the measurement becomes much less sensitive to the transparency of the sample itself. In practical terms, this means that the characteristic signal α can be decoupled more effectively from the background masking that would otherwise arise in strongly absorbing or scattering matrices. A further advantage of photothermal labels is that they can often support dual‐modal readout, combining visible color for qualitative assessment with infrared thermal output for more quantitative detection [132].

For metal‐based photothermal probes, the efficiency of signal conversion is often governed by localized surface plasmon resonance. This means that the photothermal response can be tuned through composition, size, and morphology in much the same way as optical plasmonic probes, with the key difference that the final signal is read thermally. To move beyond the absorption limits of single‐metal systems, bimetallic structures have been explored extensively. Huang et al. developed palladium–gold (Pd–Au) nanoplates as photothermal labels for the detection of microRNA‐21 (Figure 11E) [133]. By combining the high photothermal conversion efficiency of Pd with the favorable surface chemistry of Au, this system achieved a limit of detection of 0.094 pM and showed single‐base mismatch discrimination. In this case, the analytical advantage arises not simply from stronger absorption, but from the fact that the signal is shifted into a thermal dimension that is less affected by the optical limitations that constrain conventional colorimetric probes.

Photothermal conversion is not limited to plasmonic metal systems. Nonmetallic materials, particularly two‐dimensional transition metal dichalcogenides and certain carbon‐based structures, can also generate heat efficiently through light absorption followed by electron–phonon relaxation. In these cases, the usefulness of the label depends as much on the readout interface as on the material itself. Hao et al., for example, used ultrathin ReSe_2_ nanosheets, with thicknesses of only 10–20 nm, and coupled them to a smartphone‐based infrared thermal imaging accessory [51]. The resulting system maintained stable quantitative performance in serum‐rich samples, illustrating that thermal transduction can remain effective under conditions in which ordinary optical detection would be compromised by matrix‐induced attenuation. This example also highlights that the practical value of photothermal labels depends not only on photothermal conversion efficiency, but also on whether compact and sufficiently sensitive thermal readers can be integrated into portable devices.

Nonoptical labels are best understood as alternative physical strategies for signal extraction rather than simply as another class of probe materials. By converting binding events into electrochemical, magnetic, thermal, or other nonoptical outputs, they are especially useful for colored, turbid, opaque, or strongly scattering matrices where optical readout is unreliable. Electrochemical detection is commercially mature in cartridge‐based POCT systems, as illustrated by platforms such as Abbott i‐STAT. However, its integration into conventional lateral‐flow strip formats remains less established, where the main trade‐off is that improved matrix tolerance shifts complexity to electrode integration, reader calibration, and cartridge‐reader compatibility.

#### 5.1.3 Multimodal Signal Labels

Beyond single‐mode electrochemical or photothermal readout, multimodal labels that combine two or more signal channels within the same LFA platform are increasingly being explored (Figure 11F). Their main value lies in improving diagnostic confidence and matrix tolerance. In resource‐limited settings, one signal mode may provide a rapid visual result, while a second mode, such as SERS, fluorescence, photothermal, magnetic, or catalytic readout, can support more sensitive or quantitative analysis when instrumentation is available. In complex matrices, independent signal channels can also cross‐validate the same binding event, reducing the risk that matrix color, turbidity, autofluorescence, or nonspecific background can lead to misinterpretation.

For example, Fe_3_O_4_@MOF@Pt nanocomposites integrate magnetic enrichment with catalytic colorimetric amplification, allowing upstream target concentration and downstream signal enhancement to be coupled within one probe system (Figure 11G) [53]. This design was reported to lower the detection limit by 2280‐fold compared with conventional colloidal‐gold strips, reaching 0.5 pg mL^−^^1^. Other multimodal systems combine physically independent readouts for cross‐verification. An Au@PtOs platform reported for HER2‐positive exosome detection integrated SERS, catalytic colorimetric, and photothermal signals in a single LFA format (Figure 11H) [134]. In this case, the SERS channel provided molecular specificity, the photothermal channel improved matrix tolerance, and the colorimetric channel retained simple visual interpretability. Similar designs have combined SERS, fluorescence, and upconversion luminescence for miRNA detection, with limits of 0.76 fM by SERS and 2.3 fM by fluorescence [135]; SERS with magnetic enrichment for SARS‐CoV‐2 proteins, reaching 23 pg mL^−^^1^ for S protein and 2 pg mL^−^^1^ for NP [136]; and fluorescence with magnetic functionality for procalcitonin detection at 0.031 ng mL^−^^1^ [137]. These and other representative examples are summarized in Table 6.

**TABLE 6:** Representative multimodal labels for cross‐validated LFA signal transduction.

Signal modes	Probe design	Target	Sample matrix	Representative analytical performance	Main value	Ref.
SERS, fluorescence, upconversion	Au‐DTNB@Ag NPs with NaYF_4_:Yb^3^^+^,Er^3^^+^	miRNA‐21	Serum, saliva	0.76 fM by SERS; 2.3 fM by fluorescence	Multichannel optical validation	[135]
Colorimetric, magnetic, catalytic	Fe_3_O_4_@MIL‐100(Fe)@Pt nanocomposite	Procalcitonin	Serum	0.5 pg mL^−^^1^	Magnetic enrichment plus catalytic amplification	[53]
Colorimetric, photothermal	DMSN@PB nanozyme	PSA	Serum	Linear range 1–40 ng mL^−^^1^; LOD 0.202 ng mL^−^^1^	Enzymatic color amplification and photothermal confirmation	[138]
Colorimetric, SERS, photothermal	Au@PtOs nanoclusters with Raman reporters	HER2‐positive exosomes	Serum	460 exosomes µL^−^^1^ by thermal readout	Cross‐validation in exosome detection	[134]
SERS, magnetic	Fe_3_O_4_@Au satellite NPs with dual DTNB layers	SARS‐CoV‐2 S/NP proteins	Throat swabs	23 pg mL^−^^1^ for S protein; 2 pg mL^−^^1^ for NP	Magnetic enrichment and Raman specificity	[136]
Fluorescence, magnetic	SiO_2_@IOs@QDs composite spheres	Procalcitonin	Serum	0.031 ng mL^−^^1^	Magnetic handling with fluorescent quantification	[137]
Colorimetric, fluorescence, SERS	CFSERS triple‐mode probe	C‐reactive protein	Urine	6.31 ng mL^−^^1^	Triple‐mode cross‐validation	[139]
Colorimetric, SERS	Prussian blue‐based Raman reporter probes	Deoxynivalenol	Food extracts	4.21 pg mL^−^^1^	Food‐safety detection with dual readout	[140]
Colorimetric, photothermal	Au nanoshell NPs	Interleukin‐6	Serum, plasma	0.3 ng mL^−^^1^ by thermal readout	Visual plus thermal quantification	[141]
Colorimetric, photothermal	MoS_2_ nanoflowers	SARS‐CoV‐2 NP	Nasal swabs	48 pg mL^−^^1^ by thermal readout	Matrix‐tolerant dual‐mode readout	[142]

Despite these advantages, multimodal LFAs are not automatically superior for routine POCT. For samples that can be reliably interpreted using a single robust signal, adding multiple readout channels may increase reagent cost, complicate assay operation, and weaken the simplicity that makes LFAs attractive. Their main value is therefore most evident when cross‐validation or matrix tolerance justifies the added complexity. A practical concern is reader fragmentation, as many current multimodal systems still require separate devices, such as smartphone cameras, infrared thermal imagers, Raman spectrometers, fluorescence readers, or magnetic detectors, to access different signal outputs from the same strip. Multimodal labels are therefore best viewed as a systems‐integration strategy rather than simply another form of signal amplification: their translational value depends less on how many signal modes can be integrated into one nanoprobe, and more on whether those channels can be acquired, aligned, and interpreted through a coherent readout workflow.

### 5.2 Integrated Readout Platforms

Although probe engineering can substantially improve the intrinsic transduction coefficient α, the final performance of an LFA still depends on how effectively weak signals are extracted in real testing environments. This becomes particularly important for signal mechanisms that rely on specific excitation bands or highly localized transduction processes, such as SERS, UCNPs, photothermal probes, and magnetic or electrochemical systems. In such cases, the physical instability of the detection interface, stray ambient light, optical misalignment, and device‐dependent fluctuations can all contribute appreciably to *N_noise_*, even when the probe itself performs well. For this reason, the development of readout hardware can be viewed as a second layer of Phase III engineering: its purpose is to create a controlled optoelectronic, thermodynamic, or electronic microenvironment in which the signal generated by the strip can be extracted more reproducibly. As summarized in Table 7, current LFA reader architectures broadly follow three directions: dedicated desktop platforms, smartphone‐based systems, and wearable or body‐integrated formats.

**TABLE 7:** Representative integrated readout platforms for LFA signal acquisition.

Signal type	Platform format	Device dimensions	Target	Sample	Representative performance	Main integration feature	Ref.
Fluorescence	Smartphone‐based	170 × 100 × 115 mm	IgE	Serum	8.5 ng mL^−^^1^	Smartphone imaging with optical attachment	[143]
Photothermal	Smartphone‐based	47 × 84 × 118 mm	Cardiac troponin I	Serum	5.5 pg mL^−^^1^	Portable laser and thermal camera module	[79]
Ratiometric fluorescence	Smartphone‐based	140 × 75 × 55 mm	H‐FABP	Serum	0.21 ng mL^−^^1^	Ratiometric optical correction	[113]
Upconversion luminescence	Smartphone‐based	120 × 80 × 60 mm	KIM‐1/NGAL	Urine	0.28 ng mL^−^^1^ for KIM‐1; 0.23 ng mL^−^^1^ for NGAL	NIR excitation and phone‐based imaging	[128]
Upconversion luminescence	Benchtop	102 × 120 × 74 mm	PCT	Plasma	0.5 ng mL^−^^1^	Miniaturized UCL optical reader	[144]
Photothermal	Benchtop	165 × 240 × 120 mm	hCG, HIV, SARS‐CoV‐2 NP	Nasopharyngeal swabs	0.1 ng mL^−^^1^ for SARS‐CoV‐2 NP	Enclosed thermal readout system	[145]
SERS	Benchtop	137 × 210 × 280 mm	SARS‐CoV‐2	Nasopharyngeal swabs	100 copies mL^−^^1^	Raster scanning Raman reader	[146]
Magnetic flux	Benchtop	∼100 × 200 × 300 mm	hCG, G17	Serum	0.0098 mIU mL^−^^1^ for hCG; 9.1016 pg mL^−^^1^ for G17	Magnetic signal acquisition	[147]
Colorimetric	Wearable	38 × 15 × 3 mm	K^+^	Sweat	Not reported	Sweat collection and colorimetric readout	[114]
Fluorescence	Wearable	Not specified	SARS‐CoV‐2 RBD/NP; CRP proof‐of‐concept	Complex biosamples; N95‐mask bioaerosol collection	6.9 ng mL^−^^1^ for RBD; 7.2 ng mL^−^^1^ for NP; 5.5 ng mL^−^^1^ for CRP	N95‐mask bioaerosol collection with AIEgen fluorescence readout	[148]
Colorimetric	Wearable	Not specified	CEA, CA‐125, CRP	Whole blood / menstrual blood	0.85 ng mL^−^^1^ for CEA; 2.57 U mL^−^^1^ for CA‐125; 0.15 µg mL^−^^1^ for CRP	Hygiene‐pad‐integrated LFA	[149]

#### 5.2.1 Benchtop Platforms

The increasing demand for quantitative precision and reproducibility has made dedicated readers increasingly important. The limitations of unaided visual inspection are well known: human interpretation is subjective, ambient illumination is highly variable, and weak signals near the visual threshold are especially vulnerable to inter‐operator differences. Benchtop platforms address these issues by establishing an enclosed and standardized readout environment in which excitation geometry, collection optics, and environmental interference can be more tightly controlled. This reduces variability and allows the extracted signal to correlate more faithfully with analyte concentration, bringing paper‐based assays closer to laboratory‐style quantitative performance.

The engineering logic of benchtop readers is often application‐specific. Instead of shrinking a full laboratory spectrometer or microscope, many systems retain only the optical elements needed for the relevant probe. In a typical architecture, the readout chain consists of an excitation source, filters, focusing optics, and an imaging or spectroscopic detector (Figure 12A). Because the emission characteristics of the probe are predefined, broad‐spectrum scanning can often be replaced with narrow‐band collection targeted to the assay. A representative example is the miniaturized upconversion luminescence reader developed by Guo et al. for procalcitonin detection in plasma [144]. In this system, background‐free emission from UCNPs@mSiO_2_ was combined with a compact hardware layout using a high‐speed CMOS camera, dedicated bandpass optics, and an STM32 microcontroller instead of a conventional benchtop spectrophotometer (Figure 12B). The resulting device supported quantitative detection in a compact and portable format, illustrating how application‐oriented optical simplification can improve portability without sacrificing measurement quality.

**FIGURE 12:**
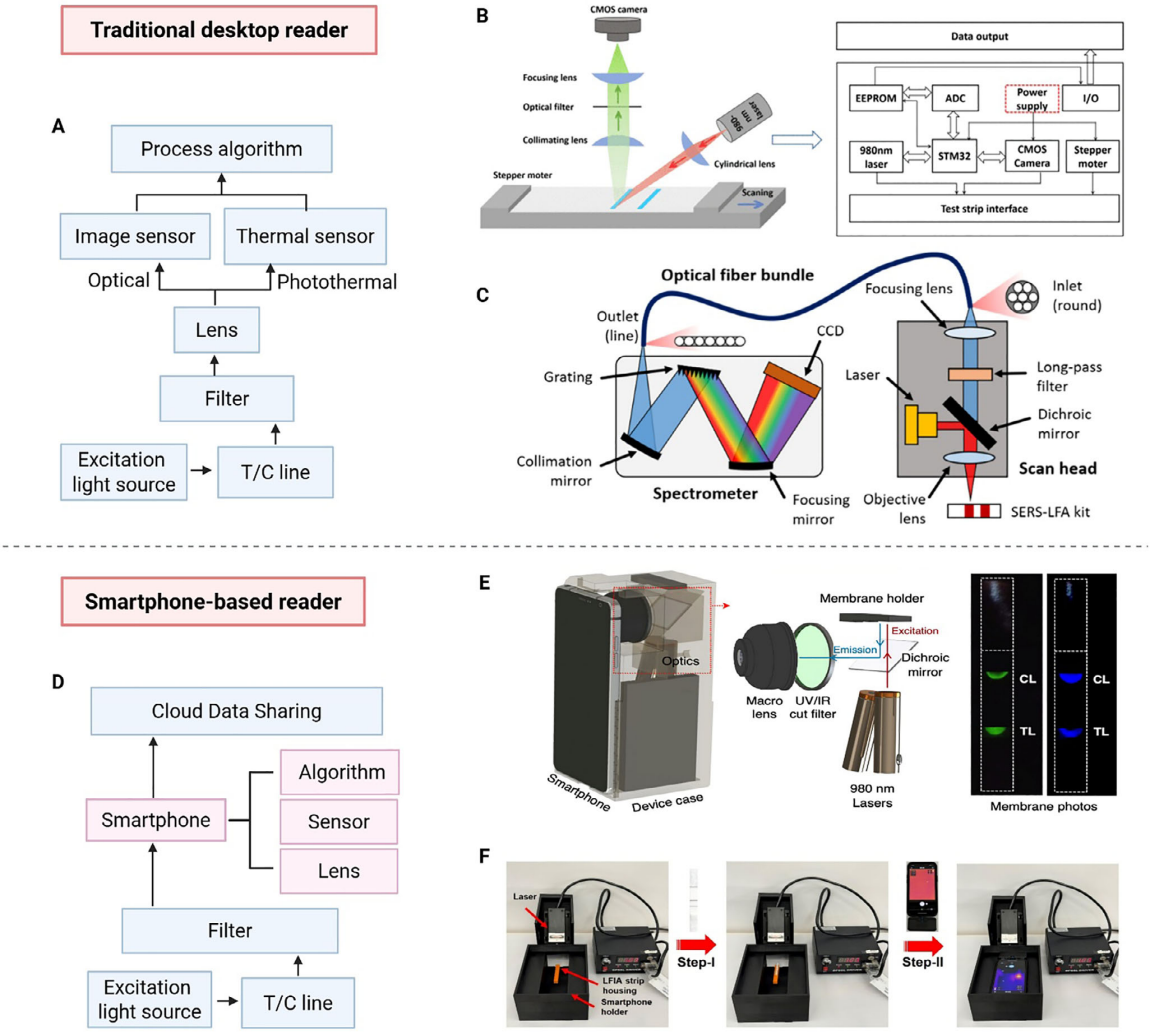
Hardware architectures for integrated LFA signal acquisition. (A–C) Benchtop readout platforms. (A) Schematic overview of signal extraction pathways in dedicated benchtop optical and photothermal LFA readers. (B) Miniaturized upconversion luminescence (UCL) reader showing the excitation, transmission, collection, and stepper‐motor scanning pathways used for UCL signal acquisition. Reproduced with permission from Ref. [144]. Copyright 2021, IEEE. (C) Optical schematic of a SERS‐LFA reader. The system utilizes a 638 nm diode laser beam and a motorized stage to perform high‐throughput grid scanning across the LFA strip at 10 µm intervals, physically smoothing signal fluctuations. Reproduced with permission from Ref. [150]. Copyright 2022, ACS. (D–F) Smartphone platforms. (D) Schematic overview of signal extraction pathways using smartphone‐based reader modules. (E) Optomechanical design of a smartphone peripheral for UCNP readout, integrating dual 980 nm lasers, a dichroic mirror, a UV/IR cut filter, and a macro lens to enable excitation and collection beyond native phone‐camera capability. Photographs show distinct UCNP emission channels on LFA membrane sensors for KIM‐1 and NGAL detection. Reproduced with permission from Ref. [128]. Copyright 2024, ACS. (F) Smartphone‐based photothermal sensing module, in which a 3D‐printed enclosure integrates a portable laser source with a plug‐in infrared thermal camera for nonoptical thermal signal extraction. Reproduced with permission from Ref. [79]. Copyright 2024, Elsevier.

Benchtop readers are also valuable when the main source of variability lies in the spatial heterogeneity of the strip itself rather than in illumination alone. Beyond the simplification and isolation of optical elements, controlled mechanical architectures in benchtop devices can effectively smooth out physical fluctuations (*N_noise_*) caused by the heterogeneous spatial distribution of nanoprobes within the porous membrane. This is especially relevant for SERS‐based LFAs, where signal intensity depends strongly on the local distribution of enhancement hotspots. A single static measurement can therefore give misleading results if the sampled region is not representative of the entire test line. Joung et al. developed a portable SERS‐LFA reader equipped with a 638 nm diode laser and a two‐dimensional motorized scanning stage (Figure 12C) [150]. Instead of acquiring a single‐point spectrum, the system performs grid‐based raster scanning across the test line at 10 µm intervals and spatially averages the resulting Raman spectra. This changes the readout logic from single‐point interrogation to area‐integrated measurement, thereby reducing noise associated with the heterogeneous distribution of nanoparticles and hotspots. In clinical testing for SARS‐CoV‐2, this strategy improved robustness and reduced false‐negative behavior. More generally, this example shows that reader design can improve quantitative performance not only by better optics, but also by physically averaging the variability of the membrane itself.

#### 5.2.2 Smartphone Platform

Smartphones have emerged as one of the most actively explored reader formats because they combine imaging hardware, local computation, and connectivity in a widely available platform. For conventional colorimetric strips, the native camera may already be sufficient for basic signal capture. However, once the assay moves into higher‐order transduction modes, such as upconversion luminescence or photothermal readout, the physical limits of the unmodified smartphone optics become more obvious. In these cases, smartphone‐based platforms usually rely on external hardware modules that extend the sensing boundary of the phone and generally function as computational hubs coupled to external modules that provide the missing excitation, filtering, or thermal sensing capability (Figure 12D).

Several recent studies illustrate this modular design logic. Arai et al. developed a smartphone‐based UCNP reader in which dual 980 nm lasers, a dichroic mirror, a UV/IR cut filter, and a macro lens were integrated into a compact accessory mounted in front of the phone camera (Figure 12E) [128]. This setup compensated for the absence of dedicated excitation and spectral filtering in the native smartphone hardware and enabled dual‐channel urine analysis of KIM‐1 and NGAL, with limits of 0.28 and 0.23 ng mL^−^^1^, respectively. Similarly, Atta et al. integrated a portable laser source and a plug‐in infrared thermal imaging module into a 3D‐printed enclosure to support smartphone‐based photothermal LFA readout (Figure 12F) [79]. In serum analysis, this platform detected cardiac troponin I down to 5.5 pg mL^−^^1^. These examples show that smartphone platforms are most effective not when they are treated as stand‐alone sensors, but when they are used as adaptable computational hubs coupled to task‐specific physical peripherals.

Even with dedicated attachments, smartphones remain non‐specialized sensors and introduce additional sources of readout variability. Lens distortion, device‐dependent auto‐exposure, variable illumination, and nonstandard viewing angles can all increase readout variability when raw strip images are interpreted directly. Software‐based calibration has therefore become central to smartphone LFA readout. In practice, preprocessing steps such as strip localization, region‐of‐interest definition, geometric normalization, and signal standardization are needed before quantitative analysis. More advanced models can then extend this calibrated image‐processing layer from correction to interpretation. In the RAD‐LFA framework developed by Du et al. [151], convolutional neural networks were used to analyze normalized strip images directly, enabling nonlinear mapping of signal features and reducing quantification bias under low signal‐to‐noise conditions. This approach achieved a coefficient of determination of R^2^ = 0.9985. Together, these developments show that smartphone‐based LFA platforms increasingly depend on coordinated optimization of optics, acquisition geometry, and algorithms, rather than on camera hardware alone.

Smartphones also have considerable potential as platforms for cloud‐based data sharing and epidemiological monitoring. Bermejo‐Peláez et al. developed a smartphone‐based reading tool integrated with AI and a cloud web platform [152]. This system enables objective, automated interpretation of various LFA test strips under diverse smartphone models and lighting conditions, synchronizing test results with a cloud database in real time, together with geographical locations and timestamps. This digitally connected model not only reduces data omissions or subjective biases caused by manual recording but also provides public health agencies with a data‐sharing platform for real‐time epidemiological tracking and case distribution monitoring. This strategy, combining local AI analysis with cloud connectivity, broadens the potential application scope of LFAs in public health surveillance and digital healthcare networks.

#### 5.2.3 Wearable Platforms

A third direction in reader development extends beyond discrete strip readout and moves toward wearable or body‐integrated systems. In these architectures, the key change lies not only in how the signal is measured, but also in how the sample reaches the strip. Conventional LFAs are based on discrete sample input: a defined volume is added, chromatography proceeds, and the signal is read after a finite time. Wearable formats alter these boundary conditions by coupling the porous sensing structure directly to body‐fluid interfaces such as skin or respiratory flow, thereby enabling continuous or semicontinuous sample collection over extended periods. This is particularly relevant for analytes that are released gradually or intermittently, such as sweat electrolytes or aerosolized pathogens. In such cases, the cumulative signal integrated over time may be more informative than a single snapshot measurement.

One challenge in wearable LFAs is that passive body‐fluid collection is much less controlled than pipetted sampling. Sweat rate, for example, can vary substantially with activity, physiology, and local skin conditions, introducing hydrodynamic fluctuations before the analyte even reaches the reaction zone. Recent wearable LFA designs therefore increasingly combine sampling control with signal readout. A representative example is the wearable paper‐based LFA for cortisol monitoring [153] (Figure 13A). This system integrates iontophoretic sweat induction, paper‐based microfluidic routing, plasmonic gold‐nanoflower colorimetric assays, and electrochromic timing modules. Rather than simply collecting sweat continuously, the device divides the sampling process into temporally resolved measurement windows, allowing sweat cortisol to be monitored under circadian, stress‐related, and jet‐lag‐associated conditions. This example shows that wearable LFAs can convert an uncontrolled skin‐fluid interface into a more structured sampling system, in which fluid generation, transport, reaction, timing, and readout are coordinated within the same epidermal platform.

**FIGURE 13:**
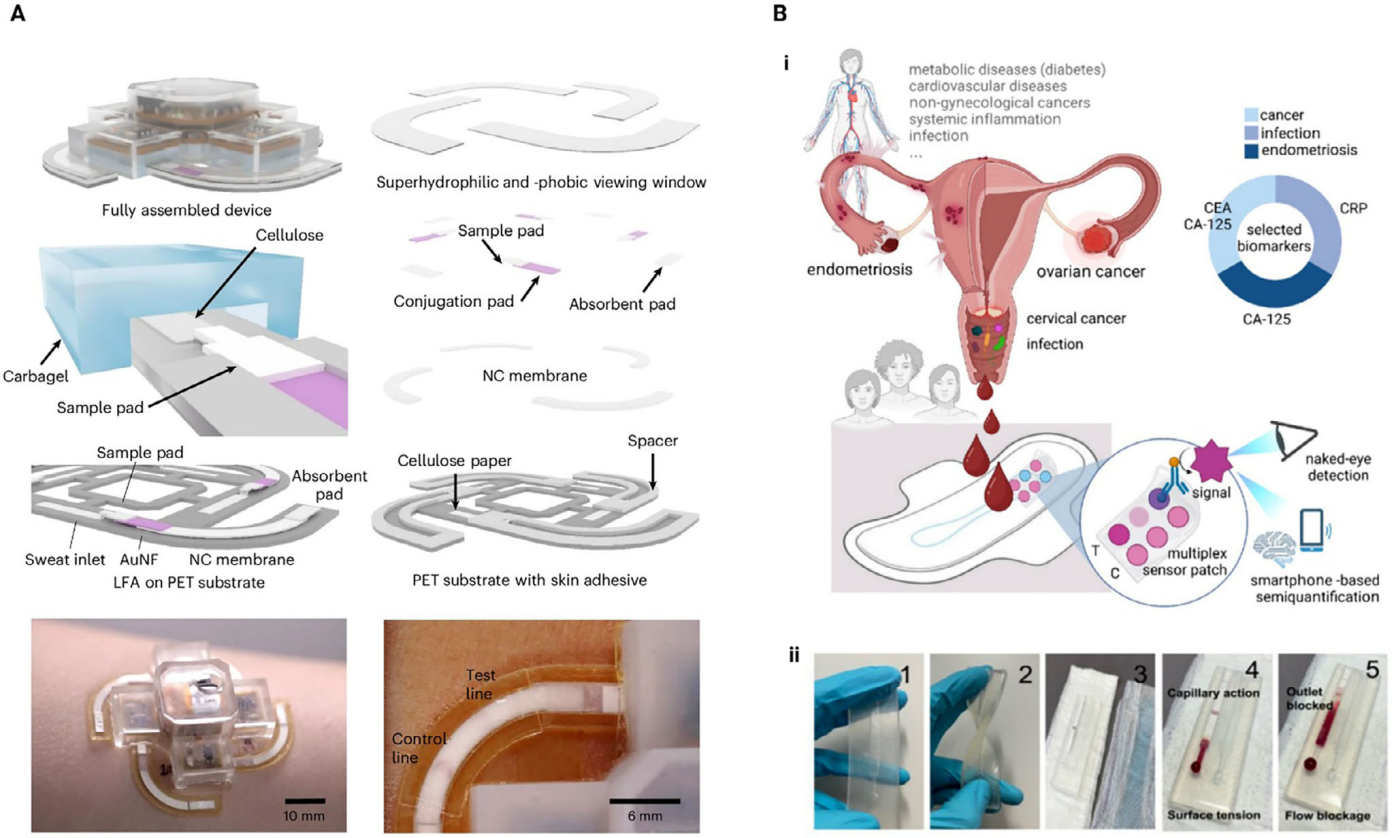
Wearable and in situ LFA platforms for body‐fluid sensing. (A) Wearable paper‐based LFA for time‐dynamic cortisol monitoring in eccrine sweat. The platform integrates iontophoretic sweat induction, paper‐based microfluidic routing, plasmonic gold‐nanoflower colorimetric assays, and electrochromic timing modules to support sequential sweat sampling and long‐term monitoring of circadian, stress‐related, and jet‐lag‐associated cortisol variations. Adapted with permission from Ref. [153]. Copyright 2026, Springer Nature. (B) Menstrual blood biosensor integrated into hygiene pads. (i) Conceptual design of the in situ LFA platform for semiquantitative biomarker analysis in unprocessed menstrual blood. (ii) Operational principle of the prototype device, including soft‐silicone casing, capillary‐based volume control, pressure‐gradient regulation, and chromatographic signal development. Reproduced with permission from Ref. [149]. Copyright 2025, Wiley‐VCH.

Wearable architectures can also exploit time integration to compensate for low analyte abundance or intermittent biomarker release. By extending the effective sampling and reaction window from minutes to hours, these systems allow weak or episodic signals to accumulate to detectable levels. The sanitary‐pad‐integrated biosensor illustrates this principle in a different body‐fluid context (Figure 13B) [149]. Instead of treating menstrual blood as a manually collected specimen, this system repurposes the multilayer absorbent structure of a commercial hygiene pad as part of the sampling and chromatographic interface. Integrated LFA sensors enabled semiquantitative detection of menstrual‐blood biomarkers over a 4 h wear period, including CEA, CA‐125, and CRP, with reported limits of detection of 0.85 ng mL^−^^1^, 2.57 U mL^−^^1^, and 0.15 µg mL^−^^1^, respectively. This example broadens the wearable LFA concept beyond sweat analysis and shows that existing wearable products can be redesigned as body‐fluid‐specific diagnostic platforms with minimal user intervention.

Although wearable formats enable continuous fluid sampling over time, current LFAs still rely on classical antibody binding with very low dissociation rates. As a result, once interfacial complexes form and color develops, the process is macroscopically irreversible. Existing wearable LFAs are therefore largely unidirectional in signal development, functioning as integrative cumulative sensors rather than systems that can reversibly track decreasing target concentrations through real‐time dynamic equilibrium, as continuous glucose monitors do. Because of this kinetic limitation, the present systems‐level value of wearable LFAs lies mainly in threshold‐based or cumulative exposure detection, such as detecting threshold crossings of environmental toxin exposure, rather than true reversible continuous monitoring. Achieving bidirectional dynamic monitoring within the LFA architecture will likely require reconstruction of the interfacial reaction network, for example through reversible competitive binding systems with controlled dissociation rates or conformationally adaptive aptamer switches.

Overall, these developments show that the readout platform should be regarded as an active determinant of LFA performance rather than a passive downstream accessory. Benchtop readers improve quantitative fidelity by tightly controlling optical or mechanical acquisition conditions, smartphone‐based platforms extend portability by combining modular sensing with local computation and connectivity, and wearable architectures broaden the temporal and physiological boundaries of sampling itself. In all three cases, the central aim is similar: to reduce the contribution of uncontrolled acquisition to *N_noise_* while retaining enough simplicity and portability for point‐of‐care deployment. This also explains why Phase III increasingly extends beyond probe chemistry alone. Once the assay approaches its practical detection limit, the quality of the reader can determine whether the information generated upstream is fully recovered or partially lost. For that reason, integrated readout platforms provide a natural transition from signal transduction to the algorithmic post‐processing strategies discussed in the next section.

### 5.3 AI/ML‐Driven Signal Processing

With the rapid development of computer vision, edge computing, and deep neural networks, AI/ML is becoming an increasingly important design‐enabling tool in LFAs [33]. Its most mature application currently lies in Phase III signal processing, where it provides a computational route for recovering analytical information that remains partially obscured by residual background, spatial heterogeneity, device‐dependent acquisition variability, and temporal signal fluctuations. In this sense, part of the burden of improving signal quality can be shifted from front‐end physical engineering to back‐end digital processing. Rather than relying only on clearer bands or more stable readers, AI‐assisted approaches aim to extract weak, distorted, or nonlinear features from imperfect raw data. This shift is gradually transforming the LFA reader from a simple acquisition device into a coupled sensing‐and‐inference system. Although the present section focuses on signal interpretation, the preceding discussions of membrane‐wicking prediction and recognition‐element screening illustrate how AI/ML can also inform Phase I and Phase II design decisions.

As summarized in Table 8, current models span several functional levels, including spatial localization and preprocessing, quantitative regression and classification, spatiotemporal kinetic prediction, multiplexed signal demodulation, and explainability modules intended to improve trust in model decisions. These methods differ substantially in their data requirements, computational cost, tolerance to noise, and practical deployment barriers. For example, lightweight models such as Random Forest or XGBoost are often attractive for mobile terminals because they perform well on modest datasets and can be deployed with limited hardware overhead [154], whereas deep convolutional architectures are more suitable when weak features must be extracted directly from raw images under highly variable acquisition conditions [155]. Time‐series models such as Long Short‐Term Memory (LSTM) or Transformer‐based networks extend this logic further by moving analysis beyond static endpoint images and into the dynamic evolution of strip signals during chromatography [112]. In this sense, AI/ML in LFAs is not a single method but a layered computational toolkit that operates at different points in the readout workflow.

**TABLE 8:** AI/ML approaches for LFA signal processing, quantification, and interpretation.

Functional role	Representative algorithm	Application in LFAs	Typical input	Main advantage	Key limitation/translational concern
Spatial localization and preprocessing	YOLO series	T/C‐line detection; ROI cropping in complex backgrounds	Raw smartphone images under variable lighting or angles	Fast detection; suitable for mobile deployment	Requires annotated training data; performance may vary across strip formats
Image segmentation	U‐Net, Mask R‐CNN	Pixel‐level background removal; weak‐signal isolation	High‐resolution cropped ROI images	Precise segmentation of heterogeneous or defective bands	Higher computational cost; risk of overfitting to specific datasets
Quantitative regression	Random Forest, XGBoost	Lightweight quantitative prediction	Manually extracted RGB, HSV, contrast, or texture features	Performs well on small datasets; low hardware requirement	Depends strongly on feature engineering and calibration transferability
End‐to‐end deep quantification	CNN, ResNet	Direct quantification from normalized strip images	ROI pixel matrices	Captures nonlinear visual features; reduces manual feature design	Requires larger datasets and external validation
Spatiotemporal kinetic prediction	LSTM, Transformer, CNN‐LSTM	Early endpoint prediction; kinetic monitoring; hook‐effect mitigation	Time‐lapse images or video streams	Shortens assay time; extracts dynamic information before endpoint	Requires continuous image acquisition and temporal calibration
Multiplex signal demodulation	ANN, SVM	Resolving cross‐reactive or overlapping multichannel signals	Spectral, colorimetric, or multimodal feature arrays	Models nonlinear relationships in multiplexed assays	Generalizability may be limited by matrix and batch variation
Clinical trust and interpretability	Explainable AI, Grad‐CAM	Visualizing decision‐relevant regions	Raw images and trained models	Improves transparency and supports regulatory discussion	Does not fully eliminate black‐box behavior

For relatively simple tasks with reasonably uniform backgrounds, conventional machine‐learning models can still provide lightweight quantitative analysis from manually extracted optical features, as discussed by Davis and Tomitaka [154]. These models are useful background examples, but their dependence on feature engineering makes them less suitable for variable matrices, spectral overlap, or cross‐reactivity. ANN‐based analysis of dual‐mode colorimetric–SERS mycotoxin signals further illustrates how nonlinear models can fuse multidimensional features for classification [140]. These two examples provide context for AI‐assisted LFAs; Figure 14 instead focuses on two representative directions that are more central to Phase III engineering: endpoint deep learning and kinetic prediction.

**FIGURE 14:**
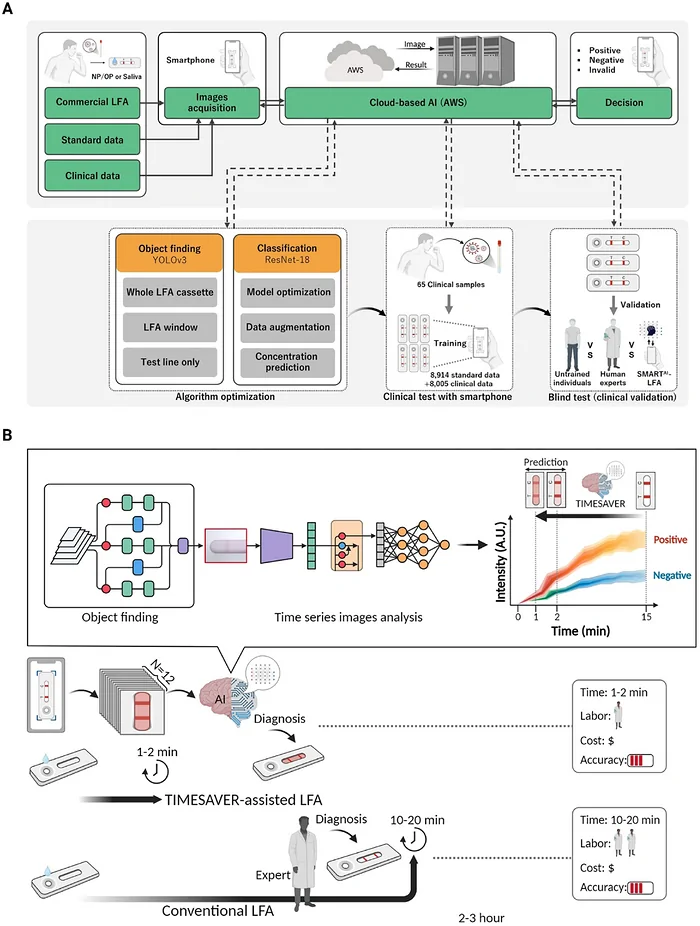
AI and machine‐learning architectures for LFA signal processing, classification and kinetic prediction. (A) SMARTAI‐LFA for deep‐learning‐assisted smartphone interpretation of SARS‐CoV‐2 antigen LFAs. The platform uses YOLOv3‐based object detection and ResNet‐18‐based classification to extract weak colorimetric signals from smartphone images, improving nucleocapsid protein detection to an LOD of 0.156 ng mL^−^^1^ and achieving 98% accuracy in smartphone‐app‐based clinical testing. Adapted under the terms of the Creative Commons Attribution 4.0 License from Ref. [156]. Copyright 2023, Springer Nature. (B) TIMESAVER framework for early kinetic prediction of LFA results. YOLO‐based ROI detection, CNN‐LSTM time‐series feature extraction, and fully connected classification allow diagnostic prediction from early color‐development sequences, enabling COVID‐19 antigen LFA readout within 1–2 min and outperforming conventional 15 min human interpretation in clinical blind testing. Adapted under the terms of the Creative Commons Attribution 4.0 License from Ref. [112]. Copyright 2024, Springer Nature.

For endpoint interpretation, deep learning can reduce the ambiguity of weak visual bands and decrease dependence on user expertise. A representative example is the SMARTAI‐LFA platform reported for SARS‐CoV‐2 antigen testing [156] (Figure 14A). This system combined smartphone imaging with a two‐step deep‐learning workflow, using YOLOv3 for object detection and ResNet‐18 for classification. The model was trained using SARS‐CoV‐2 nucleocapsid protein standard data and then retrained with clinical samples, allowing the smartphone‐based assay to provide automated positive, negative, or invalid classifications without an external cradle. Compared with untrained individuals and human experts, SMARTAI‐LFA improved the LOD for nucleocapsid protein to 0.156 ng mL^−^^1^, relative to 1.25 ng mL^−^^1^ and 0.625 ng mL^−^^1^, respectively. In smartphone‐app‐based clinical testing across different users and phones, the system achieved 98% accuracy. This example shows that AI can improve Phase III performance not by changing the chemistry of the strip, but by extracting more reliable information from faint and variable colorimetric signals.

A second challenge is generalization across different commercial test formats. Many LFA readers perform well only for a fixed cassette design, membrane geometry, or image‐acquisition condition. This limits their practical utility because real‐world POCT involves different manufacturers, strip layouts, lighting environments, and user behaviors. AutoAdapt POC addressed this issue by integrating automated membrane extraction, self‐supervised feature learning, and few‐shot adaptation for smartphone‐based interpretation of point‐of‐care diagnostic tests [157]. A base model trained on one LFA kit could be adapted to five different COVID‐19 antigen or antibody tests using only 20 labeled images per new kit. Across 726 tests, the adapted models achieved 99–100% accuracy, and in a drive‐through self‐testing study with untrained users, both antigen and antibody test kits were interpreted with 100% classification accuracy. This work highlights an important transition in AI‐assisted LFA readout: the goal is no longer simply to achieve high accuracy on one optimized assay, but to enable scalable adaptation across heterogeneous commercial tests.

The role of AI is also beginning to extend from static spatial analysis into the temporal dimension of LFA kinetics. Conventional strips are usually interpreted only after capillary flow has ceased, and the system has reached a macroscopic steady state, which often requires 15–20 min. This waiting time reflects the underlying time constants of mass transfer and interfacial reaction. Recent work has shown, however, that these constraints can sometimes be partially bypassed if early‐time image sequences are analyzed with suitable predictive models. In the TIMESAVER framework, for example, a CNN‐LSTM architecture was used to process continuous video data collected during the early stages of chromatography (Figure 14B) [112]. By learning the relationship between early signal trajectories and final endpoint values, the model was able to predict the final steady‐state result within 1–2 min after the assay began. Conceptually, this is important because it shows that AI can do more than clean up images; it can also use early kinetic information to shorten the effective readout time. This approach also connects naturally to the earlier discussion of hook‐effect mitigation, since temporal models may help distinguish misleading endpoint behavior from informative early‐stage kinetics.

Nevertheless, AI‐assisted LFAs face challenges beyond algorithmic accuracy. Deep‐learning models can behave as black boxes, complicating interpretation, regulation, and trust. Their performance may also decline under lighting changes, damaged cassettes, batch variation, unusual matrices, or rare low‐titre samples. Explainable tools, including saliency maps and Grad‐CAM, can help verify whether models focus on test and control lines rather than background artefacts. AI should therefore complement, not replace, robust assay chemistry, standardized imaging and built‐in quality control. Within the current LFA literature, AI/ML most often functions as a computational layer of Phase III signal engineering. Whereas stronger probes and improved readers mainly increase signal intensity and reduce acquisition noise, AI can extract weak spatial or kinetic features from residual distortion and variability. Its translational value will depend on external validation across devices, users, matrices, and assay formats, together with transparent reporting of training data, failure modes, and decision logic.

### 5.4 Multiplexed Signal Encoding and Decoding

As LFAs expand from single‐analyte screening to diagnostic profiling, multiplex detection has become a key extension of signal engineering. This need arises because clinically and environmentally relevant targets often differ in abundance, disease‐stage relevance, temporal dynamics, and matrix interference. Multiplexed LFAs therefore seek to read several independent signals within one assay, while maintaining sufficient spatial, spectral, or colorimetric separation for reliable decoding. Current strategies are commonly grouped into multi‐line, multi‐strip, and multi‐label architectures (Figure 15A–C and Table 9). In multiplexed LFAs, biochemical cross‐reactivity and signal crosstalk should be distinguished. Biochemical cross‐reactivity arises from unintended binding between non‐target analytes and capture or detection reagents, and is therefore primarily controlled through the specificity of the recognition system. Signal crosstalk instead arises from overlap among spectral, color, or other encoded readout channels, and is addressed through probe encoding, filtering, reader hardware, and computational decoding.

**FIGURE 15:**
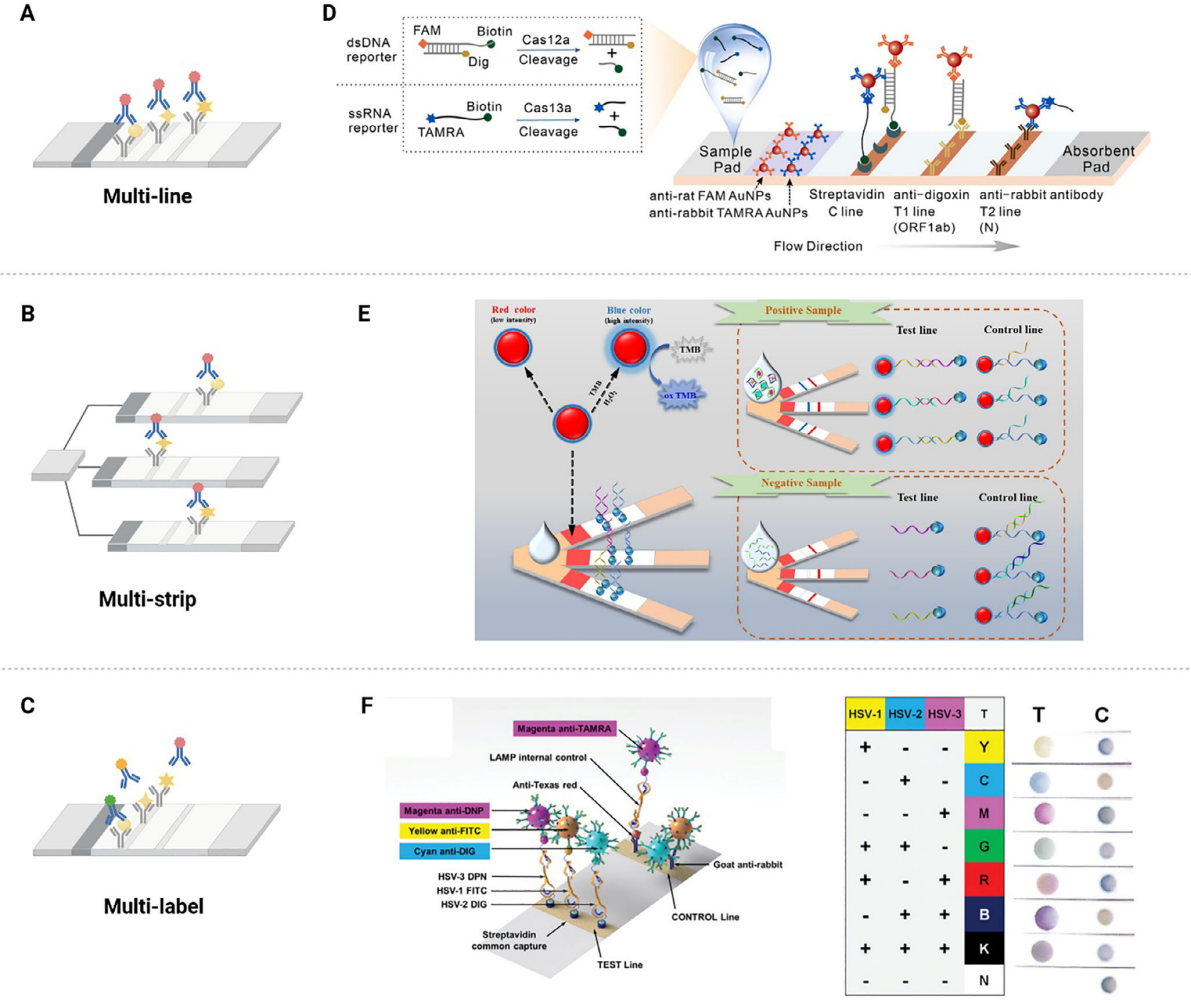
Multiplexed signal encoding and decoding strategies in LFAs. (A) Multi‐line strategy, in which multiple capture lines are arranged serially along a single chromatographic channel. (B) Multi‐strip strategy, in which the sample is divided into parallel channels to support independent reactions and reduce target depletion. (C) Multi‐label strategy, in which multiple targets are encoded within the same reaction space using probes with distinguishable optical, spectral, or colorimetric signatures. (D) Multi‐line CRISPR‐Cas LFA for simultaneous detection of dual SARS‐CoV‐2 targets using serially arranged test lines. Reproduced with permission from Ref. [158]. Copyright 2022, Elsevier. (E) Multi‐strip LFA for simultaneous detection of three mycotoxins. The parallel‐channel design reduces quantitative interference caused by upstream target depletion. Reproduced with permission from Ref. [165]. Copyright 2024, Elsevier. (F) Multi‐label LFA for herpes simplex virus subtyping using color‐encoded nanoparticles. Distinct nanoparticle colors and mixed readout patterns enable in situ decoding of multiple viral DNA profiles at a single test spot. Reproduced with permission from Ref. [164]. Copyright 2022, Wiley‐VCH.

**TABLE 9:** Representative multiplexing strategies in LFAs and their engineering trade‐offs.

Multiplexing strategy	Sample matrix	Representative targets and performance	Main advantage	Main limitation	Ref.
Multi‐line	Nasopharyngeal swabs	SARS‐CoV‐2 ORF1ab and N gene; 10 copies test^−^^1^ for both targets	Simple structure; compatible with conventional strip format	Position‐dependent target and probe depletion	[158]
Multi‐line	Cereals	DON at 0.1 ng mL^−^^1^; ZEN and AFB1 at 0.05 ng mL^−^^1^	Broad target compatibility	Increasing the number of lines complicates interpretation	[111]
Multi‐line	Urine	*Pseudomonas aeruginosa* at 12 cells mL^−^^1^; *E. coli* O157:H7 at 16 cells mL^−^^1^	Sensitive dual‐pathogen screening	Biochemical cross‐reactivity and serial depletion	[159]
Multi‐line	Urine, blood	Four‐pathogen panel at 9 cells mL^−^^1^ each	Suitable for pathogen panel screening	Biochemical cross‐reactivity and serial depletion	[160]
Multi‐line	Complex samples	H3N2 at 2 pg mL^−^^1^; SARS‐CoV‐2 at 7 pg mL^−^^1^	Rapid respiratory‐virus screening	Quantitative imbalance across lines	[161]
Multi‐strip	Clinical respiratory samples	SARS‐CoV‐2 at 0.01 ng mL^−^^1^; IAV at 0.05 ng mL^−^^1^; IBV at 0.31 ng mL^−^^1^; ADV at 0.40 ng mL^−^^1^	Reduced competition between targets	More complex fluidic design	[124]
Multi‐strip	Serum	miRNA‐21, miRNA‐20a, miRNA‐222, and miRNA‐223; 10 pM each	Better channel‐level quantitative balance	Requires controlled sample splitting	[162]
Multi‐label	Serum	CK‐MB at 0.93 pg mL^−^^1^; cTnI at 0.89 pg mL^−^^1^; myoglobin at 4.2 pg mL^−^^1^	High information density in one reaction space	Requires signal demodulation	[163]
Multi‐label	DNA samples	HSV subtype identification using color‐encoded nanoparticle combinations	Avoids serial depletion; intuitive color encoding	Color mixing and decoding complexity	[164]

The multi‐line strategy is the most straightforward format, in which several capture lines are arranged sequentially along a single flow path. Its main advantage is structural simplicity: the conventional strip format is retained while information content is expanded. This approach has been used for multiplexed nucleic acid, pathogen, respiratory‐virus, and food‐safety detection. For example, orthogonal CRISPR‐Cas systems have been integrated into a multi‐line LFA for simultaneous detection of SARS‐CoV‐2 ORF1ab and N‐gene targets, reaching 10 copies test^−^^1^ for both targets (Figure 15D) [158]. Colored cellulose nanoparticle LFAs further enabled multiplex mycotoxin detection in cereals, including DON at 0.1 ng mL^−^^1^ and ZEN and AFB1 at 0.05 ng mL^−^^1^ [111]. Similar multi‐line pathogen architectures detected 12 cells mL^−^^1^ for *Pseudomonas aeruginosa* and 16 cells mL^−^^1^ for *E. coli* O157:H7 in urine [159], and 9 cells mL^−^^1^ each for a four‐pathogen panel in urine and blood [160]. Respiratory‐virus multiplexing has also been demonstrated for H3N2 and SARS‐CoV‐2 in saliva, with photothermal LODs of 2 and 7 pg mL^−^^1^, respectively [161]. However, the main limitation of the multi‐line format is position‐dependent depletion. Because the sample encounters upstream capture zones before downstream ones, analytes and labeled probes may be partially consumed before reaching later lines. This can introduce quantitative bias along the flow direction, making multi‐line LFAs more suitable for qualitative or semiquantitative screening than for highly balanced multiplex quantification.

The multi‐strip strategy reduces this serial‐depletion problem by dividing the sample into several parallel channels or reaction units. In this format, each channel receives a fraction of the sample and performs an independent reaction, thereby providing a more comparable transport and reaction history for each target. This architecture has been used in multichannel aptamer‐based LFAs (Figure 15E) for mycotoxin detection [165], and in respiratory‐virus (0.01 ng mL^−^^1^ for SARS‐CoV‐2, 0.05 ng mL^−^^1^ for IAV, 0.31 ng mL^−^^1^ for IBV, and 0.40 ng mL^−^^1^ for ADV) [124] or microRNA panels (10 pM detections for each of miRNA‐21, miRNA‐20a, miRNA‐222, and miRNA‐223) [162]. Compared with multi‐line designs, multi‐strip platforms offer improved quantitative balance and reduced competition among capture zones. Their trade‐off is greater device complexity, because sample splitting, channel isolation, reagent loading, and flow synchronization must be carefully controlled. As a result, multi‐strip LFAs are particularly useful when balanced multiplex quantification is more important than maintaining the simplest possible strip architecture.

The multi‐label strategy achieves multiplexing through the signal probes themselves rather than through spatial separation. Different targets are encoded using probes with distinguishable colors, emission spectra, Raman fingerprints, magnetic responses, or other physical signatures. This format avoids position‐dependent depletion and allows multiple targets to be decoded within the same physical reaction zone. For example, spectrally separated probes have been used for highly sensitive cardiac biomarker detection, reaching 0.93 pg mL^−^^1^ for CK‐MB, 0.89 pg mL^−^^1^ for cTnI, and 4.2 pg mL^−^^1^ for myoglobin in serum [163]. Color‐encoded Au/Ag nanoparticles have also been used for herpes simplex virus subtyping, where different structural colors were combined and digitally decoded at a single test spot (Figure 15F) [164]. The main challenge of multi‐label multiplexing is signal demodulation. As the number of targets increases, spectral overlap, color mixing, cross‐reactivity, and reader requirements become more demanding. Therefore, multi‐label strategies often depend strongly on optical filtering, spectroscopic readout, or computational decoding.

Overall, multiplexed LFAs show that Phase III signal engineering is inseparable from the full assay workflow. Multiplex design must balance information density against transport, reaction, and hardware constraints. Multi‐line formats risk target or probe depletion; multi‐strip layouts improve reaction balance but complicate fluidics; and multi‐label schemes increase density while demanding signal separation and decoding. Effective systems therefore require co‐design of fluidics, specificity, signal encoding, and reader interpretation. Future LFAs may combine spatial and spectral encoding with AI‐assisted decoding, especially for weak or overlapping signals in complex matrices, but practical value will depend on preserving the cost, speed, and usability of LFA‐based POCT.

## 6 Conclusion

LFAs remain one of the most widely adopted point‐of‐care testing formats because they offer an unusual combination of low manufacturing cost, portability, and operational simplicity. In this review, recent advances in LFA engineering have been discussed through a three‐phase framework comprising mass transfer, reaction, and signal transduction. Organizing the field in this way helps clarify that improvements in analytical performance rarely arise from any single component alone. Instead, they reflect coordinated interventions across fluid transport, interfacial recognition, signal conversion, and data interpretation. Viewed from this systems perspective, the recent development of LFAs can be understood not only as a series of material innovations, but also as a gradual rethinking of the assay workflow.

Several broad trends emerge from this perspective. In Phase I, fluidic restructuring and membrane‐level engineering show that efficient analyte delivery to the reaction zone remains a major upstream determinant of performance, particularly for low‐abundance targets and complex matrices. In Phase II, strategies such as oriented antibody immobilization, CRISPR‐Cas‐mediated reaction amplification, and hook‐effect mitigation illustrate how the reaction stage can be redesigned to improve capture efficiency, extend dynamic range, or move beyond the stoichiometric limits of conventional immunobinding. In Phase III, advances in signal probes, integrated readers, multiplexed formats, and AI‐assisted analysis have expanded both the sensitivity and interpretability of LFA outputs by increasing signal transduction efficiency, reducing matrix‐derived background, and compensating for residual noise through hardware and software design. Although AI‐assisted analysis is currently most mature in Phase III, AI/ML can also support Phase I membrane and flow prediction and Phase II recognition‐element selection before experimental implementation. Considered together, these developments indicate that modern LFA performance is increasingly defined by coupling between phases rather than by optimization of any single phase in isolation.

At the same time, not every technically sophisticated design translates into proportionate practical value. Some strategies that are analytically attractive under laboratory conditions introduce substantial complexity in fabrication, reagent preparation, readout hardware, or workflow integration. Multimodal labels provide a useful example: although they may enable cross‐validation and richer signal extraction, multiple transduction modes can also fragment backend readout, increase reagent cost, and weaken the operational simplicity that underpins the appeal of POCT. More broadly, LFA development is unlikely to benefit from complexity for its own sake. The most useful future designs will be those that align performance gains with clearly defined clinical or field needs, rather than those that maximize technical sophistication in the abstract.

Overall, the work reviewed here suggests that LFAs continue to evolve in a direction that is both analytically ambitious and pragmatically constrained. Materials science, microfluidic engineering, signal transduction, and computational analysis are all contributing to this transition, but their value depends on whether they can be integrated without compromising manufacturability, usability, and cost‐effectiveness. The future of LFAs is unlikely to be defined by the brightest label or the most complex reader alone, but by the extent to which transport, reaction, transduction, and interpretation can be integrated into manufacturable and user‐compatible systems.

## 7 Prospects

The advances reviewed above show that LFA performance can be improved through coordinated control of transport, recognition, and signal interpretation. They also reveal a persistent gap between laboratory demonstrations and broadly deployable products. Future progress will depend on whether these strategies can be translated into manufacturable strips, simplified workflows, robust readout systems, and clinically credible use cases.

### 7.1 Manufacturable Transport Control

The Phase I strategies discussed above, including microgrooves, local geometric restructuring, engineered membrane interfaces, and passive flow barriers, can regulate residence time, analyte delivery, and reaction efficiency [69, 70, 71, 72, 73, 74, 75]. Most of these approaches nevertheless remain laboratory demonstrations or early translational concepts rather than manufacturing‐ready strip architectures. Their practical adoption remains constrained by manufacturing considerations. Fine membrane patterns may be sensitive to dimensional tolerances, while variation in pore structure or surface treatment between membrane batches can compromise flow reproducibility. Continuous processing must also remain compatible with reagent deposition, lamination, strip cutting, and package assembly. These requirements become particularly demanding when transport regulation relies on localized modifications of the membrane.

Progress in this area should therefore focus on manufacturable forms of transport control. Patterning approaches should be evaluated for their compatibility with roll‐to‐roll processing, process tolerance, and inline quality control rather than only for their ability to alter flow in a laboratory setting. Scalable surface treatments, mechanically formed structures, and integrated barriers may offer more realistic routes than highly complex architectures. Equally important, transport‐control strategies should be assessed together with reagent drying, storage stability, and lot‐to‐lot consistency. The relevant goal is not simply to delay or accelerate flow, but to obtain reproducible transport behavior without sacrificing the simplicity and cost advantages of conventional LFA production.

### 7.2 Integrated Reaction Amplification and Sample‐to‐Answer Workflows

For low‐abundance targets, the reaction‐stage advances described in Section 4 indicate that improved sensitivity rarely arises from a single amplification element. CRISPR‐Cas systems, isothermal amplification, nanozymes, and advanced signal labels can strengthen detection [52, 77, 84, 86, 87, 92, 93], but they also introduce additional reagents, reaction conditions, and timing requirements. The central translational challenge is therefore to retain the analytical benefit of these approaches while preserving the short, intuitive workflow expected of an LFA.

Future systems should integrate amplification chemistry with sample preparation, target recognition, and readout from the outset. Lyophilized reagents, spatially separated reaction zones, and sequentially rehydrated components may reduce manual mixing and incubation steps. At the same time, these integrated formats must maintain reagent activity during storage, limit unintended reactions during transport, and tolerate variation in minimally processed samples. Progress should be judged by whether a sensitivity gain remains available under a realistic sample‐to‐answer workflow, rather than by the lowest limit of detection obtained under highly controlled conditions.

### 7.3 Reader Intelligence and Explainable AI

The Phase III approaches reviewed above show that reader hardware and AI‐assisted analysis can recover useful information from faint, heterogeneous, or time‐dependent signals [112, 144, 150, 151, 156, 157]. Their clinical translation will nevertheless depend on robustness beyond proof‐of‐concept performance. Models must remain reliable across smartphone models, illumination conditions, membrane and reagent batches, sample matrices, and user behaviors. They must also operate within a defined acquisition and calibration framework, because algorithmic performance cannot compensate indefinitely for poorly controlled assay chemistry or image quality.

The next generation of reader platforms will therefore require co‐design of the strip, the acquisition hardware, and the analytical model. Standardized illumination, embedded reference features, quality‐control checks, and edge‐based inference can reduce variation at the point of use. External validation across devices, users, matrices, and test formats will be essential before clinical deployment. Explainable outputs will also be important, particularly when an algorithm flags an invalid result, a weak positive signal, or a result requiring repeat testing. AI should be regarded as a layer for signal extraction, quality assurance, and decision support, rather than as a replacement for robust chemistry, stable hardware, or transparent validation.

### 7.4 From Cumulative Wearable Strips to Dynamic Monitoring

Wearable LFA formats extend porous assays from discrete testing toward prolonged contact with body fluids such as sweat, respiratory condensate, or menstrual fluid [149, 153]. Their present strength lies in cumulative sampling, which can improve the detection of intermittent exposure, gradual biomarker release, or low‐abundance targets [149, 153]. However, most current systems remain cumulative exposure sensors rather than reversible monitors. Once a high‐affinity recognition event has produced an accumulated test‐line signal, the strip generally cannot report a subsequent decrease in analyte concentration.

Moving toward dynamic monitoring will require changes in reaction design as well as device format. Reversible competitive‐binding systems, conformationally responsive aptamer elements, and reaction networks with controlled association and dissociation kinetics may provide possible routes. These concepts will also require stable calibration over extended wear, resistance to biofouling, and an interpretable relationship between accumulated or reversible signal and analyte concentration. The goal is not to label every wearable strip as continuous monitoring, but to distinguish clearly between cumulative exposure assessment and genuinely time‐resolved measurement.

Taken together, the next stage of LFA development will depend on coordinated progress across the full assay system. Transport control must become manufacturable, reaction amplification must remain compatible with simplified operation, and intelligent readout must be validated under realistic conditions. Addressing these linked requirements can help preserve the accessibility of LFAs while extending their utility in sensitive, quantitative, connected, and longitudinal diagnostics.

## Author Contributions

Y.P. and J.L. conceived the review. Y.P. and J.L. conducted the literature search, curated the source material, and prepared the original draft. J.L., Y.L., C.M., Z.Y., L.M., and S.L. contributed to reviewing and editing the manuscript. J.L. provided supervision and project administration. All authors read and approved the final version of the manuscript.

## Conflicts of Interest

The authors declare no conflicts of interest.

## Data Availability

The data that support the findings of this study are available from the corresponding author upon reasonable request.
